# Metabolic profiling of human follicular fluid to unravel the pathways involved in polycystic ovary syndrome – a systematic review

**DOI:** 10.1007/s11154-026-10055-4

**Published:** 2026-07-06

**Authors:** Mafalda V. Moreira, Bárbara Guerra-Carvalho, Emídio Vale-Fernandes, Duarte Pignatelli, Raquel L. Bernardino, Mariana P. Monteiro

**Affiliations:** 1 Unit for Multidisciplinary Research in Biomedicine, School of Medicine and Biomedical Sciences, University of Porto, Rua Jorge Viterbo Ferreira, 228, Porto, 4050-313 Portugal; 2 i3S - Instituto de Investigação E Inovação Em Saúde, Universidade Do Porto, Porto, Portugal; 3 LAQV-REQUIMTE, Department of Chemistry, University of Aveiro, Aveiro, Portugal; 4 ITR - Laboratory for Integrative and Translational Research in Population Health, 4050-600, Rua das Taipas 135, Porto, Portugal; 5 Centre for Medically Assisted Procreation/Public Gamete Bank, Gynecology Department, Centro Materno-Infantil Do Norte Dr. Albino Aroso (CMIN), Centro Hospitalar Universitário de Santo António (CHUdSA), Unidade Local de Saúde de Santo António (ULSSA), Porto, Portugal; 6 Department of Endocrinology, Centro Hospitalar e Universitário de S. João, Porto, Portugal; 7 Department of Biomedicine, Faculty of Medicine, University of Porto, Porto, Portugal

**Keywords:** PCOS, Follicular fluid microenvironment, Metabolomic profile, Metabolic pathways

## Abstract

Polycystic ovary syndrome (PCOS), an endocrine-metabolic disorder, affects women of reproductive age. Follicular fluid (FF) reflects the biochemical microenvironment surrounding the oocyte, providing valuable insight into molecular mechanisms associated with PCOS. Metabolomics enables comprehensive profiling of small-molecule metabolites allowing the identification of disease-specific metabolic signatures, dysregulated pathways, and potential diagnostic/therapeutic targets. This systematic review summarizes available evidence on the FF metabolomic profile of women with PCOS compared to normo-ovulatory controls, aiming to identify PCOS-specific metabolic signatures and dysregulated metabolic pathways. PubMed, Web of Science, and Scopus were searched up to September 2025. Eligible studies were conducted in humans, included at least one group of women with PCOS and a control group with normal ovarian function and normal-weight. Thirty-one studies were included. FF from women with PCOS exhibited consistent metabolomic signatures across analytical platforms and cohorts. The most robust differences involved extensive lipidome alterations, along with amino acid and energy metabolism disturbances. Steroidogenic profiles showed higher testosterone and androsterone sulfate levels, while progesterone, 17-hydroxyprogesterone, and pregnenolone were decreased, suggesting impaired steroidogenesis. Fatty acid alterations included increased saturated, monounsaturated, and polyunsaturated fatty acids. Phosphatidylethanolamine species, triglycerides, and prostaglandin E2 were elevated, whereas phosphatidylcholine and lysophosphatidic acid species were reduced. Among water-soluble metabolites, isoleucine, succinate, malate, glycerol, hypoxanthine, and uracil were increased, while threonine, glutamine, arginine, citrulline, and 4PY were decreased. FF metabolomics reveals a PCOS signature characterized by lipid, amino acid, and energy metabolism dysregulation, potentially contributing to follicular dysfunction. Future studies should consider PCOS phenotypic heterogeneity.

**Supplementary Information:**

The online version contains supplementary material available at 10.1007/s11154-026-10055-4.

## Introduction

Infertility is a global public health concern, affecting millions of couples worldwide. One of the major causes of decreased female fertility is polycystic ovary syndrome (PCOS), which also stands out as one of the most common endocrine disorders among women of reproductive age [1], affecting 6–13% of the population, with up to 70% of cases remaining undiagnosed [2]. PCOS is a heterogeneous condition, diagnosed according to the Rotterdam criteria (2003), by the presence of at least two of the following: (1) oligo- or anovulation (OA), typically reflected in irregular or absent menstrual cycles; (2) clinical or biochemical hyperandrogenism (HA), such as hirsutism, acne, or elevated serum androgen levels; and (3) polycystic ovarian morphology (PCOM), defined by the presence of ≥ 12 follicles in each ovary measuring 2–9 mm in diameter and/or increased ovarian volume (> 10 mL) on transvaginal ultrasound [3]. These criteria define four phenotypes – Phenotype A (OA, HA, and PCOM), Phenotype B (OA and HA), Phenotype C (HA and PCOM), and Phenotype D (OA and PCOM) – with Phenotype A presenting the highest risk for metabolic comorbidities [3]. This inevitably results in different combinations of clinical features, which makes PCOS diagnosis a clinical challenge; thus, there is ongoing debate on which features should be used as diagnostic criteria [4–7]. In this context, recent data-driven clustering approaches integrating metabolic, hormone, and clinical data have further refined PCOS subtyping, underscoring its biological diversity and providing a subtype-based risk stratification [8].

Despite being diagnosed with a focus on reproductive manifestations, PCOS is acknowledged as a systemic disorder with significant metabolic implications, although the mechanisms underlying these links are not entirely understood. Women with PCOS are at risk of obesity [9, 10], dyslipidaemia [11, 12], insulin resistance (IR) [13, 14], type 2 diabetes mellitus [15], heart disease [16], and several types of cancer [17, 18]. PCOS aetiology is complex and remains poorly understood, but current evidence suggests it arises from an interplay between genetic, metabolic, and environmental factors [19]. Normal folliculogenesis depends on the coordinated action of luteinizing hormone (LH), follicle-stimulating hormone (FSH), and local ovarian factors, including androgens and estrogens [20]. In PCOS, despite the trigger event being unknown, this coordination is frequently disrupted, leading to follicular arrest and anovulation [21]. Several interrelated mechanisms have been proposed to account for the characteristic follicular arrest and anovulation observed in PCOS [22]. Central to this disruption is altered hypothalamic–pituitary–ovarian (HPO) axis, characterized by increased frequency and amplitude of gonadotropin-releasing hormone (GnRH) pulses, which augment LH secretion relative to FSH [23]. The resulting elevated LH:FSH ratio impairs the FSH-driven selection and maturation of a dominant follicle and predisposes granulosa cells (GCs) to premature luteinization [20, 24]. Hyperandrogenism, one of the most consistent features of PCOS, characterized by elevated levels of circulating and intra-ovarian androgens, is largely attributed to intrinsic theca-cell dysfunction [25, 26]. Elevated androgens can disrupt GCs function by inhibiting proliferation and downregulating aromatase activity, thereby impairing steroidogenesis and contributing to follicular atresia. Insulin resistance, present in up to 70–80% of women with PCOS, further amplifies hyperandrogenism through synergistic stimulation of androgen production in theca cells [27] and suppression of hepatic sex hormone-binding globulin (SHBG), thereby raising free circulating androgen levels [28]. Insulin can also influence GCs function during the later stages of folliculogenesis, particularly in steroid hormone production [29].

Intra-ovarian paracrine factors also play a role in PCOS. Anti-Müllerian hormone (AMH), predominantly secreted by small pre-antral and early antral follicles, is elevated two- to threefold in women with PCOS [30, 31]. Elevated AMH levels reduce follicular sensitivity to FSH, thereby reinforcing the arrest in follicular maturation.[32]. It also inhibits ovarian aromatase activity, promoting intra-ovarian androgen accumulation [33–35]. Thus, PCOS aetiology, instead of arising from a single defect, seems to be multifactorial, and among the putative culprits are dysregulated gonadotropin signalling, primary hyperandrogenism, paracrine interactions, and often, although not invariably, insulin resistance, which converge to impair follicular development and ovulation. These abnormalities interact in a self-perpetuating cycle of hormonal and metabolic imbalances that characterize the disorder.

Follicular fluid (FF) has emerged as a valuable matrix for unravelling the molecular fingerprints of PCOS. The FF, which originates from growing antral follicles, is a complex mixture derived from plasma components that selectively permeate the blood-follicle barrier via thecal capillaries, combined with secretions from the GCs and the oocyte [36, 37]. This biomolecule-rich reservoir mirrors the dynamic ovarian microenvironment, adjusting its composition to support oocyte growth and maturation [38, 39]. The intricate biosynthesis and transport of metabolites within the FF are vital for orchestrating follicular development, steroidogenesis, and ultimately, oocyte competence [40]. Consequently, alterations in the composition of the FF may negatively impact the function of ovarian processes and compromise reproductive success [41–43]. Extensive evidence reveals that the composition of FF in PCOS depicts marked alterations when compared to those of normo-ovulatory women. These changes involve dysregulated levels of hormones [44, 45], microRNAs [46, 47], growth factors [44, 48–50], proteins [51–54], metabolites, oxidative stress, and inflammatory markers [38, 55–57]. Moreover, many of these alterations are correlated with poorer oocyte quality parameters and reproductive outcomes. FF is obtained as a by-product during oocyte retrieval for *in vitro* fertilization (IVF) treatments, making it an ethically accessible biological matrix for molecular profiling [51].

Metabolomics offers a powerful approach to explore changes within the FF, and recent advances in omics technologies have expanded our understanding of the molecular landscape of PCOS. It involves the high-throughput profiling of low molecular weight molecules (< 1500 daltons) that participate in cellular processes, i.e., the metabolites [58], providing a near-real-time snapshot of the biological status, becoming the state-of-the-art for revealing metabolic disturbances in several pathologies, including in reproductive medicine [59–62]. Untargeted metabolomics allows the survey of the global metabolome [63–65], whereas targeted metabolomics uses panels of selected metabolites, with a wide coverage and high sensitivity, to measure alterations in specific compounds of interest [39, 66, 67]. Both approaches have been applied to FF in PCOS, identifying distinct metabolic signatures in PCOS, marked by altered hormone, amino acid, lipid profiles, as well as energy-related metabolites. Together, these studies have contributed to elucidating PCOS pathophysiology by linking metabolic alterations in the follicular microenvironment with clinical features of the syndrome [68].

In this systematic review, we aimed to summarize the available evidence on the key differences in the FF metabolome of women with PCOS as compared to those of women with normal ovulatory function. By highlighting the differences within the ovarian microenvironment, we sought to identify which metabolic pathways are disrupted, enabling a deeper understanding of the molecular mechanisms that are likely to contribute to PCOS.

## Methods

### Protocol and registration

This systematic review was conducted following the guidelines of the Preferred Reporting Items for Systematic Reviews and Meta-Analyses (PRISMA) 2020 statement [69]. The protocol was registered on October 12th, 2024, in the international database of prospectively registered systematic reviews (PROSPERO) [70] and is accessible under the registration number CRD42024596432.

### Information sources and search approach

An electronic search was conducted in PubMed, Web of Science, and Scopus databases for studies published up to September 2025. The search strategy focused on the following key concepts: polycystic ovarian syndrome, follicular fluid, and metabolomics/metabolome. Medical Subject Headings in PubMed and synonyms were used to expand the search. No restrictions were imposed on language, publication date, or other filters on the initial search. The full search string used for each database is presented in Supplementary Material.

### Study design and selection criteria

**Fig. 1:**
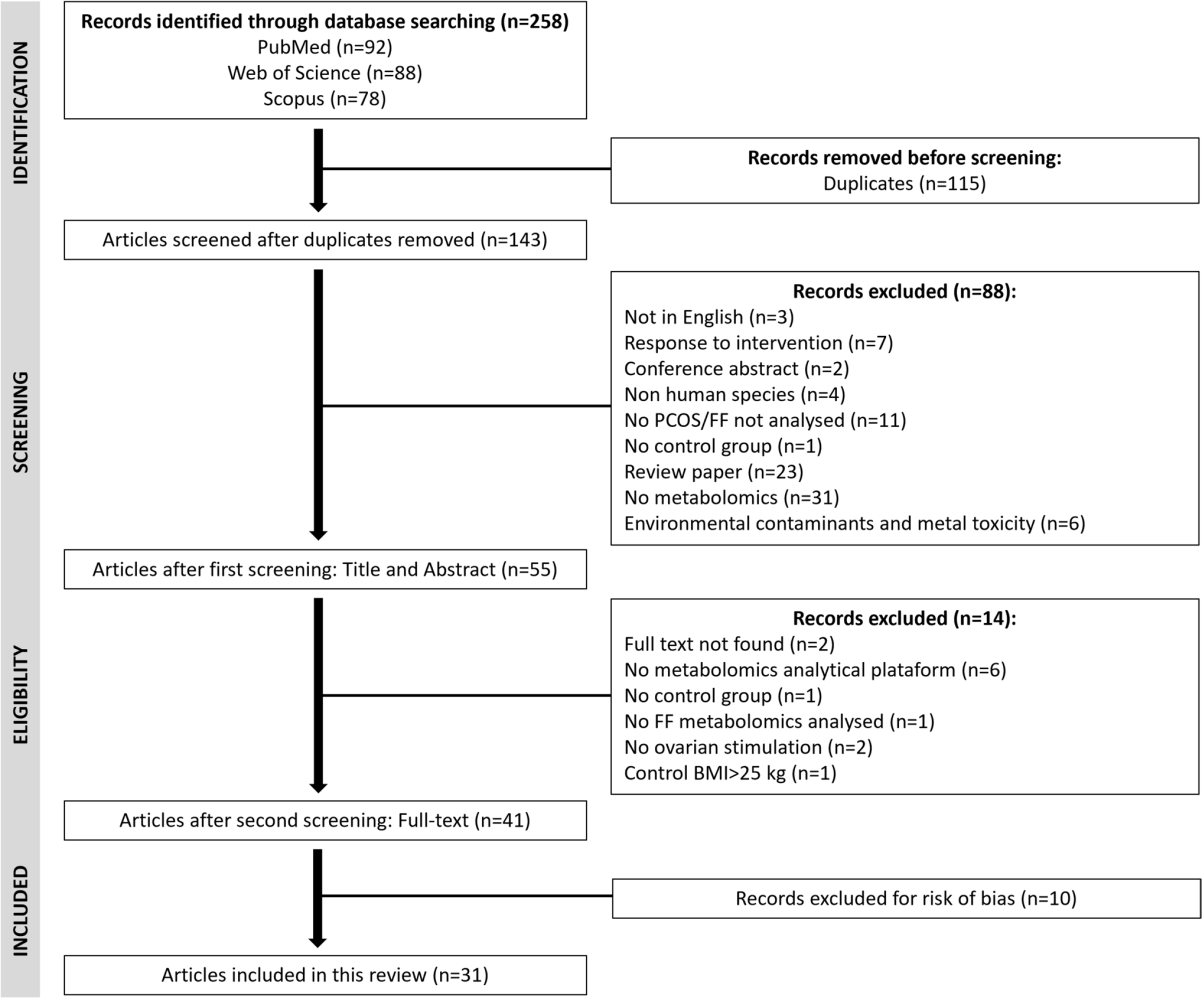
Flowchart of search and selection process for studies included in the review, adapted from PRISMA 2020 flow diagram. FF = Follicular Fluid, PCOS = Polycystic Ovary Syndrome

After removing duplicates, the study selection followed a stepwise approach. Initially, non-eligible studies were excluded based on the abstract review, followed by full-text analysis of potentially eligible studies, as outlined in detail in Fig. 1.

The eligibility inclusion criteria were based on PICOS (Population, Intervention, Control intervention, Outcome, Study design) and included observational studies (cohort, case–control, and cross-sectional studies) assessing the FF metabolomics of women with PCOS. Eligible studies were required to be conducted on humans, including at least one group of women diagnosed with PCOS and another group with normal ovarian function. Furthermore, only studies using well‑established, high‑quality analytical platforms for metabolite profiling were included, such as mass spectrometry-based techniques (GC–MS and LC–MS) or NMR spectroscopy for metabolite profiling.

The exclusion criteria comprised papers published in languages other than English, studies with no full text accessible, studies that used women with conditions that potentially impair ovarian function as controls, articles lacking a clear description of the metabolomic techniques employed, abstracts, conference proceedings, and review articles. Additionally, studies involving any form of intervention were also deemed ineligible for inclusion. To ensure optimal comparability, we included only studies that explicitly defined their control groups as women undergoing IVF primarily for male factor infertility or tubal factors of mechanical origin (post-tubal ligation or salpingectomy). Studies that reported the use of normo-ovulatory women with BMI > 25 kg/m^2^ and/or any documented history of endocrine or reproductive conditions known to alter systemic or local metabolism, as controls were excluded.

### Quality assessment

The Newcastle–Ottawa Scale (NOS) was used to assess the quality and risk of bias of the studies selected based on inclusion and exclusion criteria. Only papers that passed the quality and risk of bias assessment were included. Data from each original study included were independently extracted and checked in a cross-over manner by two independent researchers. The individual NOS quality scores for each included study are provided in Supplementary Material.

### Data extraction and synthesis

**Fig. 2:**
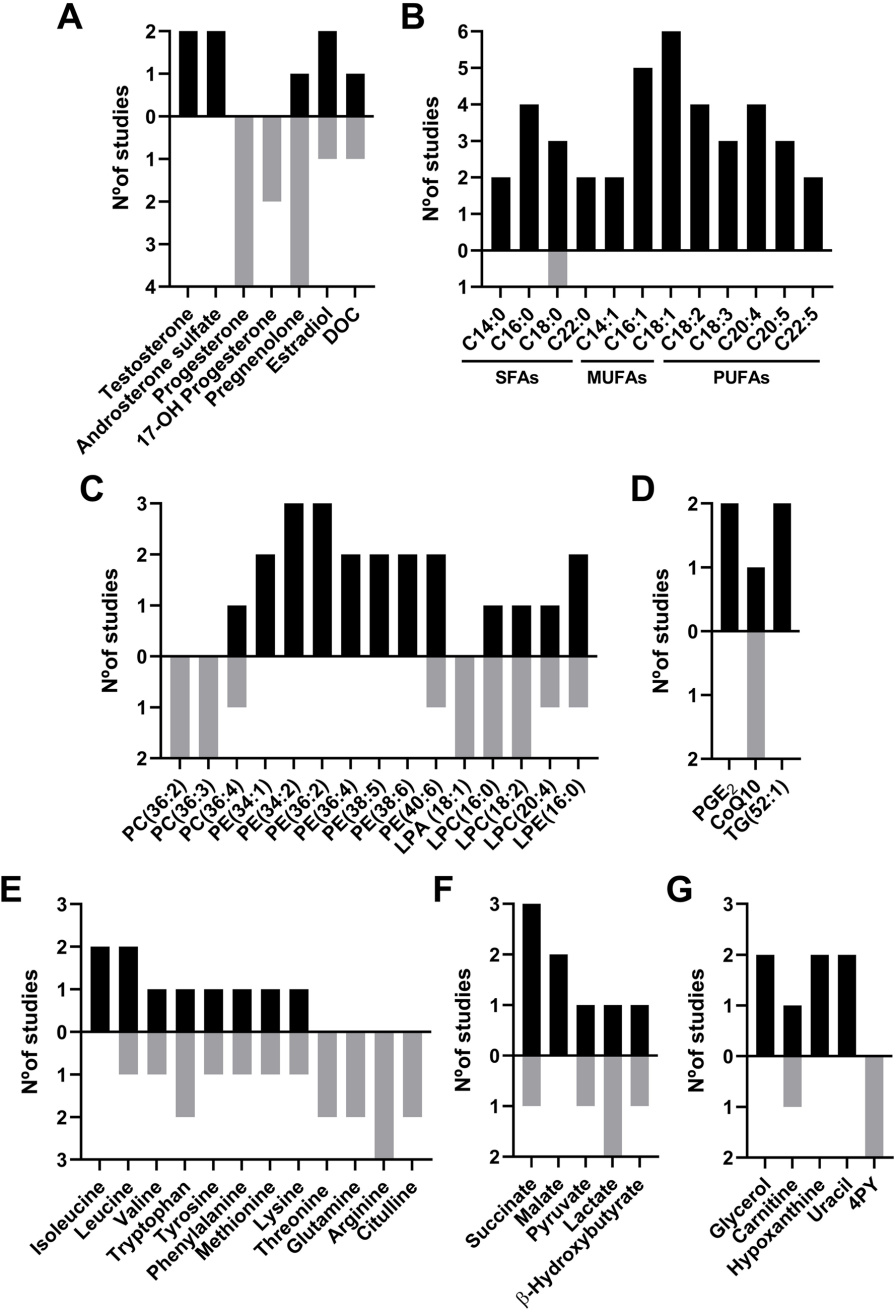
Literature-based analysis of the metabolite differences in the follicular fluid (FF) of women with PCOS compared to normo-ovulatory controls. The x-axis represents the metabolites identified as being significantly different in PCOS as compared to normo-ovulatory controls in at least two independent studies. The y-axis indicates the number of studies reporting either higher (black bars, above zero) or lower (grey bars, below zero) levels of the metabolite in the FF of women with PCOS. The metabolites were categorised into: (**A**) Steroids, (**B**) Free fatty acids (FFAs), (**C**) Phospholipids, (**D**) Other lipids, (**E**) Amino Acids, (**F**) Energy-related metabolites, and (**G**) Other water-soluble metabolites. Abbreviations: 4PY – N1-Methyl-4-pyridone-3-carboxamide; CoQ10 – Coenzyme Q 10; DOC – 11-Deoxycorticosterone; FF – Follicular fluid; FFAs – Free fatty acids; LPA – Lysophosphatidic Acid; LPC – Lysophosphatidylcholine; LPE – Lysophosphatidylethanolamine; MUFAs – Monounsaturated fatty acids; PC – Phosphatidylcholine; PCOS – Polycystic ovarian syndrome; PE – Phosphatidylethanolamine; PGE2 – Prostaglandin E2; PUFAs – Polyunsaturated fatty acids; SFAs – Saturated fatty acids; TG – Triacylglycerol

**Fig. 3:**
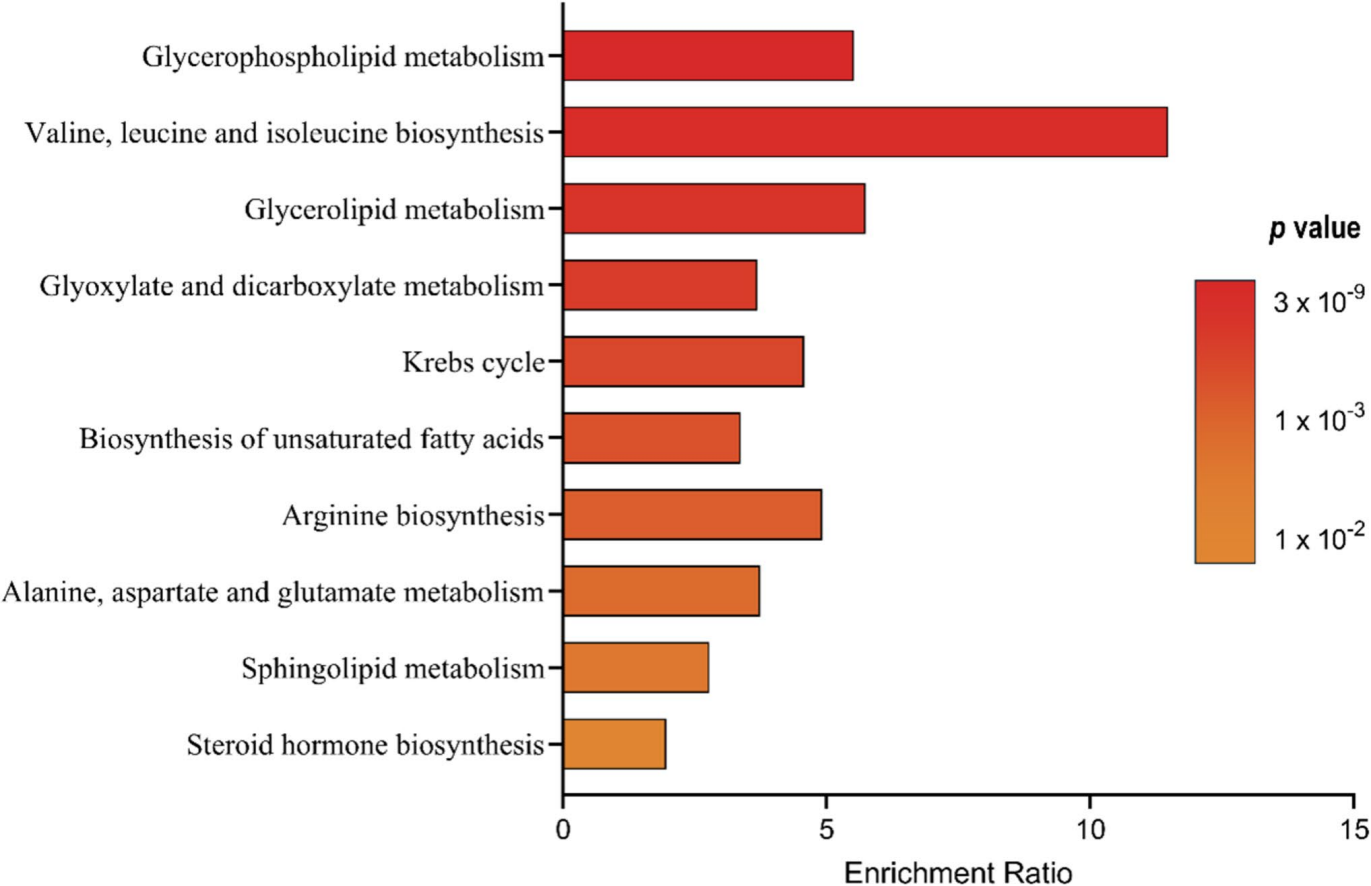
Pathway enrichment analysis of the literature-based analysis of the metabolites differently expressed in the follicular fluid (FF) of women with PCOS compared to normo-ovulatory controls. The bar plot shows the 10 top enriched metabolic pathways, with FDR-adjusted *p-*values < 0.1. The x-axis represents the enrichment ratio. The colour gradient indicates the raw *p*-value, with darker colours representing higher significance. Abbreviations: FDR – False discovery rate; FF – Follicular fluid; PCOS – Polycystic ovary syndrome

Eligible studies were independently reviewed by two authors (MVM and BGC) in a cross-over manner for data extraction, with a subsequent review conducted by a third author (RLB) to ensure consistency and accuracy. Any discrepancies between the two independent reviewers were resolved through discussion and consensus to ensure that all final inclusions and extracted data reflected mutual agreement. The data extracted included: publication details (name of the first author, year of publication, and study design), characteristics of the study population (population size, body mass index (BMI), insulin and androgens levels), method used for metabolomics analysis, and metabolites for which statistically significant differences between groups were found. The data extracted was summarised and presented in Table 1. The metabolites found to be significantly different in PCOS when compared to normo-ovulatory controls were divided into two categories: lipid and water-soluble metabolites, and a plot was created to summarize the differences (higher or lower in the FF of women with PCOS when compared to normo-ovulatory controls) in metabolite species reported to be different by at least two independent studies (Fig. 2). MetaboAnalyst 6.0 was used to perform an over-representation analysis using the enrichment analysis tool [98]. For this analysis, all the metabolites reported to be significantly different in the FF of women with PCOS compared to normo-ovulatory control women were considered. Duplicated metabolites were removed, and, whenever necessary, individual metabolites were replaced by their generic class form due to the lack of individual correspondences for all the lipid species in the KEGG database. The final metabolites list contained 263 metabolites (or metabolite classes) and is presented in Table S1 (Supplementary Material). The over-representation analysis was performed on the metabolites with matched KEGG IDs (*n* = 192) using the compound names; “metabolites” were selected as the feature type, and “KEGG (November 2025 version)” was chosen as the metabolite set library. The results are presented in Table 2 and Fig. 3.

**Table 1:** Summary of studies included in this review reporting differences in the metabolites in the follicular fluid (FF) of women with polycystic ovary syndrome (PCOS) compared to normo-ovulatory controls

First author, Publication year	Population				Study design	Method	Metabolites
	Groups	BMI (kg/m^2^)	HOMA-IR	Testosterone (nmol/L)			
Ban, Y., et al. 2021 [63]	PCOS (*n* = 8) Control (*n* = 10)	19.96 ± 1.77 20.96 ± 1.99	No information	1.84 ± 0.38 1.70 ± 0.49	Case–control	Untargeted HPLC–MS	**Higher in PCOS vs CNT:** PE(16:0/22:6); TG(16:0/14:0/18:1); TG(16:1/16:1/18:1); TG(16:0/16:0/18:2); TG(16:0/16:0/18:1); TG(16:1/18:1/18:2); TG(16:0/18:1/18:2); TG(16:0/18:1/18:2); TG(16:0/18:1/18:1); TG(18:0/16:0/18:1); TG(18:1/18:2/18:3); TG(18:2/18:2/18:2); TG(16:0/18:1/20:4); TG(18:1/18:2/18:2); TG(18:1/18:1/18:2); TG(18:0/18:1/18:2); TG(18:0/18:1/18:2); TG(16:0/18:1/20:1); Cer(d18:1/16:0); PE(16:0/18:2); PE(16:0/18:1); PE(16:0/20:4); PE(18:0/18:2); PE(18:0/18:1); PE(16:0/22:6); PE(18:1/20:4); PE(16:0/22:5); PE(18:0/20:4); PE(18:1/22:6); PE(18:0/22:6); PI(16:0/18:2); PI(16:0/18:1)
Castiglione Morelli, M.A., et al. 2019 [64]	PCOS (*n* = 12) Control (*n* = 10)	24.8 ± 5.3 22.5 ± 3.4	No information	No information	Case–control	Untargeted ^1^H-NMR	**Higher in PCOS vs CNT**: Glucose; Creatine; and Glycerol **Lower in PCOS vs CNT**: Acetate; β-Hydroxybutyrate; Leucine and Threonine
Chen, X., et al. 2020 [65]	PCOS (*n* = 35) Control (*n* = 33)	23.68 ± 3.76* 21.78 ± 3.17 *BMI higher in PCOS (*p* = 0.030)	No information	1.25 ± 0.59* 0.87 ± 0.42 *Testosterone higher in PCOS (*p* = 0.003)	Case–control	Untargeted UHPLC-MS	**Higher in PCOS vs CNT:** 2,3-Dihydroxypropyl decanoate; N-Acetylneuraminic acid; Glycerophosphocholine; Glyceraldehyde ; D-Glutamic acid; Pyridoxal 5’-phosphate; Trans-Ferulic acid; L-Carnitine; Salicylic acid; Phenylglyoxylic acid; L-Lysine; (-)-Matairesinol; 3-Methylhistidine; Purine; 1,3-Dimethyluracil; Hydroxyurea and Oxalic acid **Lower in PCOS vs CNT:** 7β-Hydroxycholesterol; Phthalic acid; Pregnenolone and 17-OH Progesterone
Chen, Y., et al. 2024 [71]	PCOS (*n* = 36) Control (*n* = 35)	23.04 ± 0.47 22.68 ± 0.62	No information	1.40 ± 0.10 1.16 ± 0.09	Case–control	Untargeted UHPLC-MS	**Higher in PCOS vs CNT:** Estrone sulfate; 2’-deoxyinosine triphosphate and L-2,4-diaminobutyric acid **Lower in PCOS vs CNT:** L-Arginine; L-Carnitine; L-Tyrosine; Progesterone; 4-trimethylammoniobutanoic acid; Succinic acid and Phytic acid
Cordeiro, F.B., et al. 2015 [72]	PCOS (*n* = 7) Control (*n* = 11)	25.5 ± 5.67 24.6 ± 2.74	No information	No information	Case–control	Untargeted LC–MS/MS	**Higher in PCOS vs CNT:** Sphingolipid (C39H79N2O6P); PC(O-16:0/15:1(9Z)) or PC(P-16:0/15:0) or PC(P-18:0/13:0) and PC (C40H72NO8P) **Lower in PCOS vs CNT:** molecule of m/z 851.3683; PS(18:3/22:6) or PS(20:4/20:5); PE (18:0/22:6); PG(17:2/22:4) or PG(19:1/20:5) or PG(22:6/17:0); PI(12:0/22:4) or PI(14:1/20:3) or PI(16:1/18:3) or PI(18:4/16:0) or PI(20:4/14:0) or PI(17:2/17:2)
Ding, Y., et al. 2022 [73]	PCOS (*n* = 25) Normal weight (*n* = 16) Obesity (*n* = 9) NonIR (*n* = 12) IR (*n* = 13) NonHA (*n* = 11) HA (*n* = 14) Control (*n* = 12)	23.77 ± 5.18 20.57 ± 1.90 29.44 ± 4.12 20.68 ± 2.08 26.61 ± 5.60 21.32 ± 3.29 25.69 ± 5.67 20.58 ± 0.91 Groups defined according to BMI	2.67 ± 1.45 1.95 ± 1.02 3.95 ± 1.21 1.49 ± 0.46 3.76 ± 1.15 2.03 ± 1.02 3.18 ± 1.57 1.23 ± 0.26 Groups defined according to HOMA-IR	1.69 ± 0.71 1.54 ± 0.63 1.96 ± 0.81 1.59 ± 0.59 1.78 ± 0.83 1.15 ± 0.62 2.12 ± 0.45 0.69 ± 0.28 Groups defined according to FAI	Case–control	Untargeted UPLC-MS	**Higher in PCOS vs CNT:** Cer(34:1;2); Cer(36:1;2); Cer(36:2;2); Cer(38:1;2); Cer(38:2;2); Cer(40:0;2); Cer(40:1;2); Cer(40:2;2); and Cer(42:1;2). C14:0; C14:1; C16:0; C16:1; C18:1; C18:3; C20:1; C20:4; C20:5; C22:0; and C22:6 **Higher in PCOS-Obesity; PCOS-IR and PCOS-HA vs CNT:** Cer(36:1;2); Cer(36:2;2); Cer(38:1;2); Cer(38:2;2;) Cer(40:0;2). C14:0; C14:1; C16:0; C16:1; C18:1; C18:3; C20:4; and C20:5 Higher in Obesity vs Normal weight PCOS: C16:0 Higher in IR vs NonIR PCOS: C16:0; C18:1; C18:3 and C20:4 Higher in HA vs NonHA PCOS**:** C16:0; C18:1; C18:3 **Lower in PCOS vs CNT:** LPG(18:0); LPG(18:1); and LPG(18:2)
Guan, S.Y., et al. 2022 [74]	PCOS (*n* = 30) Control (*n* = 30)	23.58 ± 3.83 22.71 ± 2.60	No information	1.63 ± 1.04* 0.87 ± 0.52 *Testosterone higher in PCOS (*p* = 0.030)	Case–control	Untargeted UHPLC-QE-MS	**Higher in PCOS vs CNT:** DG(15:0/18:3); DG(18:2/15:0); Androsterone sulfate and L-erythrulose **Lower in PCOS vs CNT**: LPE(16:0); L-palmitoylcarnitine; Linoleyl carnitine; trans-Hexadec-2-enoyl carnitine; 1-Arachidonoylglycerophosphoinositol; 2-propylpentanoic acid; LPA(18:1)
He, Q., et al. 2024 [75]	PCOS (*n* = 6) Control (*n* = 6)	26.83 ± 1.73* 22.08 ± 1.02 *BMI higher in PCOS (*p* = 0.040)	No information	2.26 ± 0.25* 1.30 ± 0.21 *Testosterone higher in PCOS (*p* = 0.014)	Case–control pilot study	Untargeted UHPLC-MS	**Higher in PCOS vs CNT:** PC(36:4); PC(36:5); PC(38:4); PC(38:6); PC(40:6); PC(20:4e); PC(34:1); PC(34:1); ChE(16:0); ChE(18:0); ChE(18:1); ChE(20:5); SM(d44:4); ChE(22:4); Coenzyme Q10; TG(41:2e); TG(52:1); ZyE(18:2) and DG(32:2e) **Lower in PCOS vs CNT:** PE(32:4e); ChE(19:2) and PC(36:3)
Hou, E., et al. 2021 [76]	PCOS (*n* = 32) Control (*n* = 31)	21.92 ± 0.30 21.46 ± 0.32	1.63 ± 0.09 1.52 ± 0.08	0.94 ± 0.09* 0.73 ± 0.06 *Testosterone higher in PCOS (*p* = 0.043)	Case–control	Untargeted GC–MS	**Higher in PCOS vs CNT:** Glycerol; Ethanolamine; L-Glutamate; L-Tyrosine L-aspartate; L-tryptophan; ketoleucine; L-methionine; L-isoleucine; Succinate; Guanosine; Hypoxanthine; Uridine; Uracil; D-ribose; D-glucuronic acid; D-maltose; Ribonolactone; D-threitol; C20:4; Glycerol 3-phosphate and Dopamine **Lower in PCOS vs CNT:** L-glutamine; Dimethylglycine; Pyruvate; Fumarate; Glycolic acid; Progesterone; MG(18:1) and MG(18:2)
Lai, Y., et al. 2022 [77]	PCOS (*n* = 50) Control (*n* = 50)	22.51 ± 1.82 22.18 ± 1.98	No information	0.69 (0.47)* 0.69 (0.00) *Testosterone higher in PCOS (*p* = 0.004)	Case–control	Untargeted LC–MS/MS	**Higher in PCOS vs CNT:** C18:0; C20:0; C22:0; C16:1; C18:1; C18:2; C18:3; C20:4; C20:5; C22:5 and C22:6
Lazzarino, G., et al. 2021 [67]	PCOS (*n* = 14) Control (*n* = 35)	24.8 ± 6.3 23.4 ± 5.1	No information	No information	Case–control	Targeted HPLC	**Higher in PCOS vs CNT:** Lactate; Hypoxanthine; Xanthine; β-pseudouridine; Uracil; Cytosine; Cytidine; Malondialdehyde; 8-OH-dG; Nitrite and Nitrate **Lower in PCOS vs CNT:** Ascorbate; Vitamin A; Vitamin D; Coenzyme Q10; Carotenoids; Threonine; Arginine; Valine; Methionine and Tryptophan
Li, S., et al. 2020 [78]	PCOS (*n* = 39) Control (*n* = 30)	22.98 ± 3.30 20.53 ± 2.65	2.81 ± 1.58* 1.28 ± 0.42 *HOMA-IR higher in PCOS (*p* < 0.001)	1.30 ± 0.83* 0.75 ± 0.45 *Testosterone higher in PCOS (*p* < 0.05)	Case–control	Targeted HPLC–MS	**Higher in PCOS vs CNT**: 8,9-DHET; 11,12-DHET; PGI_2_; PGE_2_; PGD_2_; PGF_2α_; TXB_2_; PGJ_2_ and 15-deoxy-Δ12,14-prostaglandin J_2_
Liu, L., et al. 2019 [79]	PCOS (*n* = 15) Control (*n* = 36)	24.8 ± 3.7* 21.6 ± 3.2 *BMI higher in PCOS (*p* = 0.007)	No information	No information	Case–control	Untargeted UPLC-MS	**Higher in PCOS vs CNT:** 5-Phenyl-1,3-pentadiyne; [6]-Dehydroshogaol; 1-Hydroxy-2,12,15-heneicosatrien-4-one; C15:0; Vitexin 6’’-(3-hydroxy-3-methylglutarate); LPC(15:0); LPC(18:2); LPC(20:2); LPC(16:0); LPC(16:1); LPC(20:4); LPE(16:0); Indan-1-ol; 2,5-Undecadienal; 5-(3’-Hydroxyphenyl)-gamma-valerolactone-3’-O-glucuronide; p-HPEA-EDA; 2-p-Tolyl-1-propene,p-Mentha-1,3,5,8-tetraene; (R)−3,4-Dihydro-2-methyl-2-(4,8,12-trimethyl-3,7,11-tridecatrienyl)−2H-1-benzopyran-6-ol; Arachisprenol 12; ar-Artemisene; beta-Calacorene; 1-Pentadecene and (Z)−1,5-Undecadiene **Lower in PCOS vs CNT:** Triethanolamine; Androstenol; Allyl benzoate; 6-tridecylsalicylic acid; (3R, 6’Z)−3,4-Dihydro-8-hydroxy-3-(6-pentadecenyl)−1H-2-benzopyran-1-one; 3’-N-Acetyl-4’-O-(14-methylheptadecanoyl)fusarochromanone; Methylmalonic acid; Lentinic acid; Neuromedin B (1–3); Lysyl-Valine; Prolyl-Methionine; VPGPR Enterostatin; Randilongin; 16-hydroxypalmitic acid; 2-Hexyl-5-[2-(4-hydroxy-3-methoxyphenyl)ethyl]furan; Malyl-CoA; Tridecanol; DG(18:0/18:4); DG(17:2/20:4); Ethyl furoate; LPA(P-16:0e); PC(22:1/P-18:0); PC(22:2/P-18:01); PC(O-18:1/16:0); PC(O-22:0/20:4); PC(O-22:2/22:3); PC(O-22:0/22:6); PGP(16:0/20:4); PGP(16:0/22:4); (S)−3,4-Dihydroxybutyric acid; 1H-Indol-3-ylacetyl-myo-inositol; (S)−2-Acetolactate; Paxilline; 25-Hydroxyvitamin D2; 2-Phenylbutyric acid; Campestanol; Lithocholic acid glycine conjugate; Ubiquinol 8; Anandamide; Adlupone; (3beta,11alpha,13beta)−3,11,13-Oleananetriol; 3-Hexaprenyl-4-hydroxybenzoic acid; Anhydrocinnzeylanol; S-Japonin; 4-Hydroxy-3-(16-methylheptadecyl)−2H-pyran-2-one; Coenzyme Q10; beta-Ionol; Cer(d18:0/16:0); Cer(d18:0/24:0); Cer(d18:1/22:0); Galabiosylceramide (d18:1/20:0); Galabiosylceramide (d18:1/24:1); Galabiosylceramide (d18:1/24:1); Ganglioside GA1 (d18:1/24:1); Ganglioside GA1 (d18:1/18:1); Glucosylceramide (d18:1/24:0); SM(d18:1/14:0); Tetrahexosylceramide (d18:1/24:0); Tetrahexosylceramide (d18:1/18:1); TG(18:4/15:0/18:4) and TG(18:4/20:5/o-18:0)
Liu, X., et al. 2024 [80]	PCOS (*n* = 52) Normal weight (*n* = 26) Overweight (*n* = 26) Control (*n* = 43) Normal weight (*n* = 30) Overweight (*n* = 13)	23.69 ± 3.77 20.67 ± 2.21 26.71 ± 2.27 22.81 ± 3.14 21.24 ± 1.89 26.44 ± 2.35 Groups defined according to BMI	No information	2.05 ± 1.28* 2.01 ± 0.94* 1.42 ± 0.80 1.25 ± 0.87 *Testosterone higher in PCOS groups (*p* < 0.05)	Case–control	Untargeted UPLC-MS	**Higher in PCOS vs CNT**: 3,4-Dihydro-6-methoxy-2,2-dimethyl-2H-1-benzopyran-4-ol **Lower in PCOS vs CNT**: Asterosterol; 3-(Dimethylaminomethyl)indole; Notoginsenoside I; Citronellyl anthranilate; and Tetrahydroaldosterone-3-glucuronide Higher in overweight PCOS vs normal weight PCOS: Ganglioside GD3 (d18:1/23:0); 2,3-Butanediol glucoside and schleicherastatin 5 Lower in overweight PCOS vs normal weight PCOS: LPC(O-18:0); LPC(22:1); LPE(22:2/0:0); LPC(17:0); LPE(20:1/0:0); PE(P-16:0e/0:0); 2′-Apo-beta-carotenal; and 3beta-Acetoxy-19alpha-hydroxy-12-ursene Higher in Overweight CNT vs normal weight CNT: LPC(18:0) Lower in Overweight CNT vs normal weight CNT: LPC(18:1)
Ma, Y., et al. 2022 [81]	PCOS (*n* = 16) Control (*n* = 16)	23.69 ± 1.75 23.04 ± 1.87	No information	2.84 ± 1.46* 1.18 ± 0.42 *Testosterone higher in PCOS (*p* < 0.01)	Case–control	Targeted GC–MS	**Higher in Overweight PCOS vs overweight CNT:** C16:0; C16:1; C18:0; C18:1; C18:2n6; MUFAs and PUFAs **Higher in Normal weight PCOS vs Normal weight CNT:** C20:4n6 and SFA **Lower in normal weight PCOS vs normal weight CNT:** PUFAs
Marie, C., et al. 2023 [82]	PCOS (*n* = 27) Control (*n* = 54)	26.10 ± 0.94* 23.70 ± 0.54 *BMI higher in PCOS (*p* = 0.0273)	No information	No information	Case–control	Targeted GC–MS	**Higher in PCOS vs CNT:** Testosterone **Lower in PCOS vs CNT**: Estradiol; Progesterone and Pregnenolone
Niu, Z., et al. 2014 [83]	PCOS (*n* = 55) Normal weight (*n* = 30) Obesity (*n* = 25) Control (*n* = 63) Normal weight (*n* = 38) Obesity (*n* = 25)	22.90 ± 3.10 32.40 ± 2.40 22.10 ± 2.80 32.90 ± 2.20 Groups defined according to BMI	1.4 ± 0.8 2.4 ± 0.9* 0.9 ± 0.5 2.0 ± 1.0 *HOMA-IR higher in PCOS obesity group (*p* < 0.05)	1.75 ± 0.8 2.04 ± 0.8* 1.53 ± 0.4 1.49 ± 0.6 *Testosterone higher in PCOS obesity group (*p* < 0.05)	Case–control	Untargeted GC–MS	**Higher in PCOS with obesity vs PCOS with normal weight and CNT:** C16:0 and C18:1n9 Higher in Obesity PCOS vs Normal-weight PCOS: C16:0 Higher in Obesity CNT vs Normal-weight CNT: C16:0
Qian, Y., et al. 2024 [84]	PCOS (*n* = 8) Control (*n* = 8)	22.42 ± 2.95 20.85 ± 1.79	No information	No information	Case–control	Untargeted LC–MS	**Higher in PCOS vs CNT:** PE(16:0/18:1); PE(14:0/22:3); PE(17:1/17:1);TG(14:0/14:0/18:2); PE(14:1/24:4); LPA(18:2);PE(18:0/18:2); TG(12:0/16:1/18:1); DG(16:0/16:0); TG(14:0/14:0/18:1); PE(20:3/20:3); TG(12:0/16:0/18:1); DG(18:1/18:1); DG(16:0/18:1); PE(18:0/22:5); TG(12:0/16:1/18:2); DG(18:1/18:2); PE(16:0/18:1); PE(14:0/22:3); PE(17:1/17:1);TG(14:0/14:0/18:2); PE(14:1/24:4); LPA(18:2);PE(18:0/18:2); TG(12:0/16:1/18:1); DG(16:0/16:0); TG(14:0/14:0/18:1); PE(20:3/20:3); TG(12:0/16:0/18:1); DAG(18:1/18:1); DG(16:0/18:1); PE(18:0/22:5); TG(12:0/16:1/18:2); DG(18:1/18:2) **Lower in PCOS vs CNT:** SHexCer(d39:2); SHexCer(d38:1); PC(20:3e/26:4); PC(14:1e/21:2); PC(16:2e/22:6); SM(d14:0/23:1); SM(d14:1/30:2); SM(d14:1/23:1); LPC(18:2); LPC(22:5); LPC(20:4); LPC(22:6); LPI(20:4); HexCer/NS(d18:1/24:1); GlcADG(20:0/20:0) and PC(16:1e/22:4)
Sallicandro, L., et al. 2024 [85]	PCOS (*n* = 9) Control (*n* = 10)	24.10 ± 2.10 24.90 ± 4.40	No information	No information	Case–control	Untargeted LC–MS	**Higher in PCOS vs CNT:** 1-O-Octadecyl-sn-glyceryl-3-phosphorylcholine (LPC(O-18:0)) **Lower in PCOS vs CNT:** Fucose-1-phosphate; LPC(18:1); 1,2-Dioleoyl-sn-glycero-3-phosphatidylcholine (PC(18:1/18:1)); PC(16:0/18:2); 1-Oleoyl-L-α-lysophosphatidic acid (LPA(18:1)); 1-Linoleoyl-sn-glycero-3-phosphorylcholine (LPC(18:2)); Arachidonoylthiophosphorylcholine (PC-S(20:4)) and LPC(16:0)
Shen, H., et al. 2022 [86]	PCOS (*n* = 28) Control (*n* = 28)	23.50 ± 3.94 22.78 ± 2.59	No information	No information	Case–control	Untargeted LC–MS/MS	**Higher in PCOS vs CNT:** 3,4-dehydrothiomorpholine-3-carboxylate; N-acetyl-S-(Nmethylcarbamoyl) cysteine; L-NIL; Umbelliferone; and Soyasaponin aa **Lower in PCOS vs CNT:** Deoxyadenosine; (E)−1-O-cinnamoyl-beta-D-glucose; 6-(2-hydroxyethoxy)−6-oxohexanoic acid; Aspartyl-lysine and Epidermin
Sun, Y., et al. 2023 [87]	PCOS (*n* = 21) Control (*n* = 27)	23.04 (21.1;26.35) 21.17 (20.4;21.94)	2.12 (1.79,2.46) 1.40 (1.22,1.58)	No information	Case–control	Untargeted GC–MS	**Higher in PCOS vs CNT:** C16:1; C18:1; cis-Vaccenic acid (18:1Δ11cis); trans-Vaccenic acid (18:1Δ11trans); Conjugated linoleic acid (18:2); 10,12-Octadecadienoic acid (C18:2); 8,11,14-Eicosatrienoic acid (C20:3n-6); C22:5; Citramalic acid; 2-Hydroxybutyric acid; Azelaic acid; Malic acid and Succinic acid **Lower in PCOS vs CNT:** C8:0; C6:0; C18:0; 3-Pentenoic acid; Ala-Ala; 1-Aminocyclopropane, NADP_NADPH
Sun, Z., et al. 2019 [88]	PCOS (*n* = 19) Control (*n* = 21)	23.9 ± 4.4 22.7 ± 2.3	No information	1.73 ± 1.70 1.25 ± 0.80	Case–control	Untargeted UPLC-MS	**Higher in PCOS vs CNT:** Deoxycorticosterone; 3-Hydroxynonanoyl carnitine; Phenylalanine; Leucine, and C20:5 **Lower in PCOS vs CNT:** LPC(14:0); LPC(16:0); LPC(18:0); and Phytosphingosine
Vale-Fernandes, E., et al. 2025 [89]	PCOS (*n* = 20) Control (*n* = 20)	24.78 ± 5.66 23.92 ± 3.28	1.68 ± 1.35 1.10 ± 0.78	No information	Case–control	Untargeted ^1^H-NMR	**Higher in PCOS vs CNT:** Citrate **Lower in PCOS vs CNT:** Formate, Lactate
Xu, Y., et al. 2024 [90]	PCOS (*n* = 12) NonHA-PCOS (*n* = 6) HA-PCOS (*n* = 6) Control (*n* = 6)	22.42 ± 5.79 21.48 ± 1.96 20.37 ± 2.23	No information	1.50 ± 0.43 1.52 ± 0.39 0.76 ± 0.23 Groups defined according to androgen levels	Case–control	Untargeted GC/MS and LC–MS	**Higher in PCOS vs CNT:** y-Tocopherol; C20:2n6; C18:2; C18:1n9; C19:1n9; C18:1n7 and C20:1n9 **Higher in HA-PCOS vs CNT:** y-Glutamylleucine**;** y-Glutamylisoleucine**;** y-Tocopherol**;** DHEA-S**;** Estradiol C19:0; C16:0; C17:0; C14:0; C18:0; C18:4n3; C18:3; C20:2n6; C22:2n6; C18:2; C18:1n9; C22:1n9; C17:1n7; C19:1n9; C12:1n7; C18:1n7; C20:1n9; C14:1n5 and C16:1n7 Higher in HA-PCOS vs NonHA-PCOS**:** Methionine; Isoleucine; Estradiol; C19:0; C16:0 2-OH; C16:0; C17:0; C14:0; C18:0; C18:4n3; C18:3n3; C22:2n6 C18:2n6; C18:1n9; C22:1n9; C17:1n7; C19:1n9; C12:1n7; C18:1n7; C20:1n9; C14:1n5; C16:1n7 and DHEA-S **Lower in PCOS vs CNT:** Citrulline and GSSG
Yang, X., et al. 2021 [91]	PCOS (*n* = 35) Control (*n* = 31)	24.29 ± 3.00 23.14 ± 2.78	No information	1.32 ± 0.77* 0.72 ± 0.35 *Testosterone higher in PCOS (*p* < 0.001)	Case–control	Targeted UPLC-MS	**Higher in PCOS vs CNT:** Glycocholic acid; Taurocholic acid; Glycochenodeoxycholic acid and Chenodeoxycholic acid-3- d-glucuronide
Yang, Z., et al. 2021 [92]	PCOS (*n* = 141) NonHA (*n* = 87) HA (*n* = 54) Control (*n* = 166)	23.18 ± 3.75* 23.92 ± 3.69* 21.32 ± 2.89 *BMI higher in PCOS (*p* < 0.05; *p* < 0.05)	No information	1.70 ± 0.54 3.09 ± 0.40 1.34 ± 0.53 Groups defined according to androgen levels	Case–control	Targeted LC–MS	**Higher in PCOS vs CNT:** Androstenedione and Testosterone **Higher in PCOS-HA vs CNT:** Estrone **Higher in PCOS-NA vs CNT:** Cortisol **Lower in PCOS vs CNT:** Pregnenolone; 17-OH Pregnenolone and 11-Deoxycorticosterone
Yu, J., et al. 2024 [93]	PCOS (*n* = 45) Control (*n* = 36)	21.65 ± 1.55 21.20 ± 1.42	No Information	No information	Case–control	Untargeted LC–MS	**Higher in PCOS vs CNT:** Pregnanolone sulfate; SM(14:1;O2/28:5); PGE_2_; Epitestosterone; LPE(20:4); LPE(22:6); LPE(22:4); LPE(22:5); PE(38:6); PE(34:2); PE(36:2); PE(36:4); LPE(18:0); LPE(16:0); Palmitoyl ethanolamide; Thymine; Androsterone sulfate; LPI(16:0); LPE(20:3); PC O-20:3; LPC(20:3); 2-Linoleoyl glycerol and; LPE(18:2) **Lower in PCOS vs CNT:** LPC O-14:0; 9-(S)-HPODE; LPI(22:6); PC O-20:0; N1-Methyl-4-pyridone-3-carboxamide; 17-OH Progesterone; Pregnenolone; Hydroxyprogesterone caproate; 2-Hydroxystearic acid and 2-Methylbutyroylcarnitine
Yu, L., et al. 2021 [94]	PCOS (*n* = 10) Control (*n* = 10)	20.84 ± 0.94 20.73 ± 0.95	No information	4.16 ± 0.45* 1.87 ± 0.31 *Testosterone higher in PCOS (*p* = 0.001)	Case–control	Targeted LC–MS	**Higher in PCOS vs CNT:** Pregnenolone; Estriol and Estradiol **Lower in PCOS vs CNT:** Progesterone
Yu, L., et al. 2025 [95]	PCOS (*n* = 79) Normal BMI (≤ 24 kg/m^2^; *n* = 57) High BMI (> 24 kg/m^2^; *n* = 22) Control (*n* = 64) Normal BMI (*n* = 48) High BMI (*n* = 16)	22.85(20.5, 25.5) 21.7(19.8, 22.5)	No information	1(0.6, 1.22)* 0.7(0.4, 1) *Testosterone higher in PCOS (*p* = 0.01)	Case–control	Targeted HPLC–MS	**Lower in PCOS vs CNT:** Glutamine; Taurine; Phenylalanine; Tryptophan; Arginine; Histidine; Serine; Citrulline; Lysine; Sarcosine and 1-Methylhistidine Lower in overweight PCOS vs normal-weight PCOS: Taurine, Alanine, Tryptophan, Methionine, Citrulline and α-Aminoadipic acid Lower in PCOS-IR vs PCOS-NonIR: Taurine, Alanine, Tryptophan, Arginine, Methionine, Citrulline, Sarcosine, and α-Aminoadipic acid
Zhang, M., et al. 2024 [96]	PCOS (*n* = 22) Control (*n* = 23)	23.20 ± 2.24* 21.53 ± 2.37 *BMI higher in PCOS (*p* = 0.019)	4.02 ± 2.22* 1.17 ± 0.31 *HOMA-IR higher in PCOS (*p* < 0.001)	1.53 ± 0.66* 1.11 ± 0.49 *Testosterone higher in PCOS (*p* = 0.022)	Case–control	Targeted UPLC-MS	**Lower in PCOS vs CNT:** DAG(38:4); PE-P(40:6); PE-P(40:5); PE-P(40:4); PE-P(38:5); PE-P(38:4); PE-P(38:3); PE-P(36:3); PE-P(36:2); PE-P(36:1); PE-P(34:2); PI(36:3); PI(36:2); PI(40:4); GM3 d18:1/24:0; PC-P(34:2); PC-P(34:1); PC-P(36:2); PC-P(38:4); PC-P(38:3); PC(36:4); PC(36:3); PC(36:2); PC(36:1); SM(d18:1/50:0); SM(d18:1/16:0); SM(d18:0/16:0); SM(d18:1/24:1); SM(d18:1/26:1) and Plasmalogen PE
Zhao, H., et al. 2015 [97]	PCOS (*n* = 95) Control (*n* = 91)	24.83 ± 0.44* 22.37 ± 0.42 *BMI higher in PCOS (*p* < 0.001)	2.71 ± 0.17* 2.07 ± 0.09 *HOMA-IR higher in PCOS (*p* = 0.008)	1.46 ± 0.10* 0.85 ± 0.03 *Testosterone higher in PCOS (*p* < 0.001)	Case–control	Targeted GC/MS and HPLC–MS	**Higher in PCOS vs CNT:** Pyruvate; Valine; Isoleucine; Leucine; α-keto-β-methylvalerate; α-ketoisovalerate; α-ketoisocaproate; β-Hydroxybutyrate; Succinate; Malate; and Oxaloacetate **Lower in PCOS vs CNT:** Lactate; acyclcarnitines Hexanoyl; Malonyl; Hydroxyisovaleryl; Octenoyl; and Adipyl; cis-aconitate; N-methylnicotinamide, N1-methyl-2-pyridone-5-carboxamide and N1-methyl-4- pyridone-3-carboxamide

Abbreviations: ^1^*H-NMR* Proton Nuclear Magnetic Resonance; *8-OH-dG* 8-hydroxy-2’-deoxyguanosine; *9(S)-HPODE* (10E,12Z)−9-Hydroperoxyoctadeca-10,12-dienoate; *BMI* Body mass index; *Cer* Ceramide; *ChE* Cholesterol ester; *CNT* Control group; *DG* Diacylglycerol; *DHEA-S* Dehydroepiandrosterone sulfate; *DHET* Dihydroxyeicosatrienoic acid; *GC–MS* Gas Chromatography-Mass Spectrometry; *GlcADG* Glucosyladipate; *GSSG* Oxidized Glutathione; *FAI* Free Androgen Index; *HA* Hyperandrogenism; *HOMA-IR* Homeostasis Model Assessment of Insulin Resistance; *HexCer/NS* Hexosylceramide; *IR* Insulin resistance; *LC–MS* Liquid Chromatography-Mass Spectrometry; *LPA* Lysophosphatidic Acid; *LPC* Lysophosphatidylcholine; *LPE* Lysophosphatidylethanolamine; *LPG* Lysophosphatidylglycerol; *MG* Monoacylglycerol; *MUFAs* Monounsaturated fatty acids; *NADP* Nicotinamide Adenine Dinucleotide Phosphate; *NADPH* Reduced Nicotinamide Adenine Dinucleotide Phosphate; *NHA* Non-hyperandrogenism; *OHSS* Ovarian hyperstimulation syndrome; *PC* Phosphatidylcholine; *PCOS* Polycystic ovary syndrome; *p-HPEA-EDA* p-hydroxyphenyl ethylamine ethyl ester; *PE* Phosphatidylethanolamine; *PG* Phosphatidylglycerol; *PGD2* Prostaglandin D2; *PGE2* Prostaglandin E2; *PGF2α* Prostaglandin F2 alpha; *PGI2* Prostacyclin; *PGJ2* Prostaglandin J2; *PGP* Phosphoglycerophosphate; *PI* Phosphatidylinositol; *PS* Phosphatidylserine; *PUFAs* Polyunsaturated fatty acids; *SHexCer* Sulfatide Hexosylceramide; *SM* Sphingomyelin; *TG* Triacylglycerol; *TXB2* Thromboxane B2; *ZyE* Zymosterol Ester

**Table 2:** Over-representation analysis results showing the enriched metabolic pathways of the metabolites reported to be significantly different in the follicular fluid of women with PCOS compared to normo-ovulatory controls

Metabolic pathway	Total number of metabolites in the pathway	Expected number of observed metabolites	Number of observed metabolites	Enrichment ratio	Raw *p*-value	FDR-adjusted *p*-value	Observed metabolites in the pathway
Glycerophospholipid metabolism	38	2.9	16	5.517241379	2.68E-09	2.17E-07	Serine, Glycerol 3-phosphate, PC, Ethanolamine, PG, PE, DG, Glycerophosphocholine, LPA, PI, PS, LPI, PGP, LPC, LPE, LPG
Valine, leucine and isoleucine biosynthesis	8	0.61	7	11.47540984	9.57E-08	3.87E-06	Leucine, α-ketoisovalerate, Valine, Threonine, Ketoleucine, Isoleucine, α-Keto-β-methylvalerate
Glycerolipid metabolism	16	1.22	7	5.737704918	8.13E-05	0.00219	Glycerol 3-phosphate, Glycerol, Fatty acid, TG, DG, LPA, MG
Glyoxylate and dicarboxylate metabolism	32	2.44	9	3.68852459	0.000407	0.00592	Pyruvate, Glutamate, Acetate, Oxaloacetate, Glutamine, Serine, Malate, Citrate, cis-Aconitate
Krebs cycle	20	1.53	7	4.575163399	0.000425	0.00592	Pyruvate, Oxaloacetate, Succinate, Fumarate, Malate, Citrate, cis-Aconitate
Biosynthesis of unsaturated fatty acids	39	2.97	10	3.367003367	0.000438	0.00592	C20:4, C16:0, C18:1, C18:0, C18:2, C20:3, C20:0, C18:3, C20:5, C22:6
Arginine biosynthesis	16	1.22	6	4.918032787	0.000739	0.00782	Glutamate, Aspartate, Arginine, Glutamine, Fumarate, Citrulline
Alanine, aspartate and glutamate metabolism	28	2.14	8	3.738317757	0.000772	0.00782	Pyruvate, Glutamate, Oxaloacetate, Succinate, Aspartate, Glutamine, Fumarate, Citrate
Sphingolipid metabolism	33	2.52	7	2.777777778	0.0101	0.0912	Serine, Cer, SM, Glucosylceramide, GM3, GA1, Phytosphingosine
Steroid hormone biosynthesis	87	6.63	13	1.960784314	0.0122	0.0985	Androstenedione, Progesterone, Estrone, Testosterone, Cortisol, Estradiol, 17-OH progesterone, Pregnenolone, Estrone sulfate, Deoxycorticosterone, DHEA-S, 17-OH Pregnenolone, Estriol
Arachidonic acid metabolism	44	3.36	8	2.380952381	0.0156	0.115	PC, C20:4, PGE2, PGF2α, PGD2, PGI2, 8,9-DHET, 11,12-DHET
Nicotinate and nicotinamide metabolism	15	1.14	4	3.50877193	0.0227	0.153	NADP, Aspartate, N-Methylnicotinamide, N1-Methyl-4-pyridone-3-carboxamide
Pyruvate metabolism	23	1.75	5	2.857142857	0.0262	0.154	Pyruvate, Acetate, Oxaloacetate, Fumarate, Malate
Valine, leucine and isoleucine degradation	40	3.05	7	2.295081967	0.0283	0.154	Leucine, α-ketoisovalerate, Valine, Ketoleucine, Isoleucine, α-Keto-β-methylvalerate, Methylmalonic acid
Histidine metabolism	16	1.22	4	3.278688525	0.0285	0.154	Glutamate, Aspartate, Histidine, Methylhistidine
Phenylalanine, tyrosine and tryptophan biosynthesis	4	0.305	2	6.557377049	0.0313	0.158	Phenylalanine, Tyrosine
Glycine, serine and threonine metabolism	33	2.52	6	2.380952381	0.0349	0.166	Pyruvate, Serine, Threonine, Sarcosine, Creatine, Dimethylglycine
Linoleic acid metabolism	5	0.381	2	5.249343832	0.0495	0.223	PC, C18:2
Pantothenate and CoA biosynthesis	20	1.53	4	2.614379085	0.06	0.256	Aspartate, Uracil, α-ketoisovalerate, Valine
alpha-Linolenic acid metabolism	13	0.991	3	3.027245207	0.0704	0.272	PC, C18:3, C18:4
Nitrogen metabolism	6	0.458	2	4.366812227	0.0706	0.272	Glutamate, Glutamine
D-Amino acid metabolism	15	1.14	3	2.631578947	0.1	0.369	Oxaloacetate, Serine, Glutamate
Butanoate metabolism	16	1.22	3	2.459016393	0.117	0.387	Glutamate, Succinate, β-Hydroxybutyrate
Phenylalanine metabolism	8	0.61	2	3.278688525	0.119	0.387	Phenylalanine, Tyrosine
Taurine and hypotaurine metabolism	8	0.61	2	3.278688525	0.119	0.387	Taurine, Taurocholic acid
One carbon pool by folate	26	1.98	4	2.02020202	0.131	0.407	Serine, Methionine, Sarcosine, Dimethylglycine
Neomycin, kanamycin and gentamicin biosynthesis	2	0.153	1	6.535947712	0.147	0.44	D-Glucose
Glutathione metabolism	28	2.14	4	1.869158879	0.16	0.462	NADPH, NADP, Glutamate, GSSG
Pyrimidine metabolism	39	2.97	5	1.683501684	0.171	0.478	Glutamine, Uracil, Thymine, Uridine, Cytidine
beta-Alanine metabolism	21	1.6	3	1.875	0.212	0.566	Aspartate, Uracil, Histidine
Inositol phosphate metabolism	32	2.44	4	1.639344262	0.223	0.566	D-Glucuronic acid, DG, PI, Phytic acid
Lysine degradation	32	2.44	4	1.639344262	0.223	0.566	Succinate, Lysine, Carnitine, 4-Trimethylammoniobutanoic acid
Arginine and proline metabolism	37	2.82	4	1.418439716	0.31	0.758	Pyruvate, Glutamate, Arginine, Creatine
Glycolysis/Gluconeogenesis	26	1.98	3	1.515151515	0.318	0.758	Pyruvate, Acetate, Oxaloacetate
Lipoic acid metabolism	40	3.05	4	1.31147541	0.364	0.843	Pyruvate, α-ketoisovalerate, Ketoleucine, α-keto-β-methylvalerate
Tyrosine metabolism	42	3.2	4	1.25	0.4	0.885	Pyruvate, Tyrosine, Fumarate, Dopamine
Starch and sucrose metabolism	18	1.37	2	1.459854015	0.404	0.885	D-Glucose, D-Maltose
GPI-anchor biosynthesis	32	2.44	3	1.229508197	0.446	0.941	C16:0, PE, PI
Ubiquinone and other terpenoid-quinone biosynthesis	20	1.53	2	1.307189542	0.458	0.941	Tyrosine, Coenzyme Q10
Primary bile acid biosynthesis	46	3.51	4	1.13960114	0.471	0.941	Taurine, Glycocholic acid, Taurocholic acid, Glycochenodeoxycholic acid
Fatty acid biosynthesis	47	3.58	4	1.117318436	0.488	0.941	C16:0, Long-chain fatty acid, C8:0, C14:0
Purine metabolism	73	5.57	6	1.077199282	0.488	0.941	Glutamine, Hypoxanthine, Xanthine, Guanosine, Deoxyadenosine, 2’-Deoxyinosine triphosphate
Cysteine and methionine metabolism	35	2.67	3	1.123595506	0.507	0.941	Pyruvate, Serine, Methionine
Vitamin B6 metabolism	9	0.686	1	1.457725948	0.511	0.941	Pyridoxal 5’-phosphate
Biotin metabolism	10	0.763	1	1.31061599	0.549	0.988	Lysine
Ascorbate and aldarate metabolism	11	0.839	1	1.191895113	0.583	1	D-Glucuronic acid
Galactose metabolism	27	2.06	2	0.970873786	0.623	1	D-Glucose, Glycerol
Pentose and glucuronate interconversions	19	1.45	1	0.689655172	0.78	1	D-Glucuronic acid
Ether lipid metabolism	20	1.53	1	0.653594771	0.797	1	Glycerophosphocholine
Fatty acid elongation	38	2.9	2	0.689655172	0.8	1	C16:0, Long-chain fatty acid
Fatty acid degradation	39	2.97	2	0.673400673	0.812	1	Fatty acid, C16:0
Fructose and mannose metabolism	21	1.6	1	0.625	0.813	1	Fucose 1-phosphate
Propanoate metabolism	22	1.68	1	0.595238095	0.827	1	Succinate
Steroid biosynthesis	41	3.13	2	0.638977636	0.834	1	Calcidiol, ChE
Pentose phosphate pathway	23	1.75	1	0.571428571	0.841	1	D-Ribose
Amino sugar and nucleotide sugar metabolism	31	2.36	1	0.423728814	0.917	1	Fucose 1-phosphate
Porphyrin metabolism	31	2.36	1	0.423728814	0.917	1	Glutamate
Tryptophan metabolism	40	3.05	1	0.327868852	0.96	1	Tryptophan
Drug metabolism—other enzymes	42	3.2	1	0.3125	0.966	1	NADP
Drug metabolism—cytochrome P450	59	4.5	1	0.222222222	0.992	1	2-Propylpentanoic acid

Abbreviations: *CoA* Coenzyme A; *Cer* Ceramide; *ChE* Cholesterol ester; *DHEA-S* Dehydroepiandrosterone sulfate; *DG* Diacylglycerol; *DHET* Dihydroxyeicosatrienoic acid; *FDR* False Discovery Rate; *GA1* Ganglioside GA1; *GM3* Ganglioside GM3; *GPI* Glycosylphosphatidylinositol; *GSSG* Oxidized glutathione; *LPA* Lysophosphatidic acid; *LPC* Lysophosphatidylcholine; *LPE* Lysophosphatidylethanolamine; *LPG* Lysophosphatidylglycerol; *LPI* Lysophosphatidylinositol; *MG* Monoacylglycerol; *NADP* Nicotinamide adenine dinucleotide phosphate; *NADPH* Reduced NADP; *PC* Phosphatidylcholine; *PCOS* Polycystic ovary syndrome; *PE* Phosphatidylethanolamine; *PG* Phosphatidylglycerol; *PGD2* Prostaglandin D2; *PGE2* Prostaglandin E2; *PGF2α* Prostaglandin F2α; *PGI2* Prostaglandin I2; *PGP* Phosphatidylglycerol phosphate; *PI* Phosphatidylinositol; *PS* Phosphatidylserine; *SM* Sphingomyelin; *TG* Triacylglycerol

## Results

### Study selection

The initial search retrieved 258 papers: 92 at PubMed, 88 at Web of Science, and 78 at Scopus. After eliminating duplicates, 143 papers were identified for analysis. Among those, 88 papers were excluded based on the information provided in the abstract, and 14 additional papers were excluded when full-text analysis was considered. The remaining 41 papers that met the eligibility criteria were subjected to risk of bias assessment, which led to the exclusion of 10 additional papers. As a result, a total of 31 papers describing studies that met the pre-defined inclusion criteria were included in the systematic review. The full text of the included studies was reviewed again in detail by two authors. The search and selection process for the studies included in the review is illustrated in the flowchart in Fig. 1.

### Summary of included studies

The characteristics of the 31 studies included in this systematic review are summarised in Table 1. This table details each study’s design, participant characteristics (BMI, HOMA-IR, and testosterone levels), analytical platform, and metabolite findings. Collectively, these studies demonstrate that FF from women with PCOS exhibits a distinct metabolomic profile when compared to normo-ovulatory controls.

### Metabolomic alterations in FF of women with PCOS

To identify robust metabolic changes, we focused on metabolites reported by at least two independent studies. The metabolites recurrently found to be different in the FF of women with PCOS were classified into two broad categories: lipids and water‑soluble metabolites.

#### Lipid metabolites

Lipids encompass a diverse array of molecules that perform critical functions within the follicular microenvironment, ranging from hormone precursors and energy reserves to membrane constituents and signalling mediators [99]. Steroid hormones derive from cholesterol and orchestrate the endocrine regulation of female reproductive function [100], while free fatty acids serve both as metabolic substrates and modulators of cellular signalling. Phospholipids form the structural matrix of cell membranes, influence membrane fluidity and receptor function, while other lipids—such as neutral lipids—participate in energy storage and inflammatory processes [99]. For a more detailed description of the lipid profile that distinguishes the FF of women with PCOS, lipid metabolites were organised into four main subclasses: steroids (Fig. 2A), fatty acids (Fig. 2B), phospholipids (Fig. 2C), and other lipids (Fig. 2D).

##### Steroids

Figure 2A illustrates a summary of the hormone profile, showing the number of articles that reported specific hormones and hormone precursors to be higher or lower in the FF of women with PCOS as compared to normo-ovulatory controls. Androgenic hormones, including testosterone [82, 92] and androsterone sulphate [74, 93], were consistently found to be higher in the FF of women with PCOS across multiple studies. These findings are aligned with the hyperandrogenism that characterises women with PCOS. In contrast, progesterone [71, 76, 82, 94] and 17-OHP4 [65, 93] were found to be consistently lower in the FF of women with PCOS, suggesting major shifts in the steroidogenic pathway and impaired follicular maturation. For pregnenolone, the results show a minor inconsistency, since four studies reported lower levels [65, 82, 92, 93], while one study found pregnenolone levels to be higher [94] in the FF of women with PCOS. Estradiol levels depicted variable results across the studies, with two studies reporting higher levels in the FF of women with PCOS [90, 94], while one study reported the contrary [82]. Finally, the results for 11-deoxycorticosterone were also inconsistent among studies, with one study reporting higher levels [88] and another reporting the contrary [92].

Overall, these findings underpin the ovarian steroidogenesis shifts towards androgen synthesis as key features of PCOS, while also disclosing the inconsistency regarding follicular estrogen synthesis. These hormonal shifts are likely to contribute to the disrupted folliculogenesis and anovulatory phenotype characteristic of this condition.

##### Free fatty acids

Figure 2B illustrates a literature-based analysis of the free fatty acid (FFA) profile in the FF of women with PCOS compared to normo-ovulatory controls, summarising the number of studies reporting significant differences in specific FFAs.

Saturated fatty acids (SFAs), C14:0 [73, 90], C16:0 [73, 81, 83, 90] and C22:0 [73, 77] were consistently reported to be higher in the FF of women with PCOS. Although with a small inter-study variability, C18:0 showed a similar tendency, with three studies reporting higher levels [77, 81, 90], while one study found lower levels [87] in the FF of women with PCOS. Similarly, monounsaturated fatty acids (MUFAs), C14:1 [73, 90], C16:1 [73, 77, 81, 87, 90], and C18:1 [73, 77, 81, 83, 87, 90] were observed to be consistently higher across multiple studies in the FF of women with PCOS, as well as polyunsaturated fatty acids (PUFAs), namely C18:2 [77, 81, 87, 90], C18:3 [73, 77, 90], C20:4 [73, 76, 77, 81], C20:5 [73, 77, 88], and C22:5 [77, 87].

Overall, the FFAs’ profiles suggest there are significant differences in the lipid profiles within the FF of women with PCOS, characterised by an accumulation of saturated and unsaturated FFAs.

##### Phospholipids

Figure 2C presents a literature-based analysis of the differences in phospholipid and lysophospholipid levels found in the FF of women with PCOS compared to normo-ovulatory controls, summarising the number of studies reporting higher or lower levels of each specific lipid species.

In the phospholipids class, several phosphatidylcholine (PC) and phosphatidylethanolamine (PE) species were identified as potential biomarkers of PCOS in the FF. Within the PC class, both PC(36:2) [85, 96] and PC(36:3) [75, 96] presented consistently lower levels in the FF of women with PCOS compared to normo-ovulatory controls. Conversely, PC(36:4) showed inconsistent results, with one study reporting higher levels [75] and another study reporting the opposite [96]. Regarding PEs, the vast majority were found to present consistently higher levels in the FF of women with PCOS, namely PE(34:1) [63, 84], PE(34:2) [63, 84, 93], PE(36:2) [63, 84, 93], PE(36:4) [63, 93], PE(38:5) [63, 84] and PE(38:6) [63, 93]. Only PE(40:6) showed some variability, with two studies reporting higher levels [63, 84] and one reporting lower levels [72] in the FF of women with PCOS.

Regarding the lysophospholipids, lysophosphatidic acid 18:1 (LPA(18:1)) was reported to be lower in the FF of women with PCOS as compared to normo-ovulatory controls by two independent studies [74, 85]. However, lysophosphatidylcholines (LPC) and lysophosphatidylethanolamines (LPE) levels were found to be inconsistent across studies. LPC (16:0) and LPC(18:2) were reported to be lower in the FF of women with PCOS in two studies ([85, 88] for LPC(16:0), [84, 85] for LPC(18:2)), while another found it to be higher [79]. Similarly, discrepant findings were observed for LPC(20:4) levels in the FF of women with PCOS, with one study reporting higher [79] and another reporting lower [84] levels, as well as for LPE(16:0), with levels found to be higher in two studies [79, 93] and lower in another one [74].

Overall, these results suggest consistent differences in several PC and PE lipid species in the FF of women with PCOS, which suggests an overall altered glycerophospholipid profile in this condition.

##### Other lipids

In addition to FFAs and glycerophospholipids, three other lipid species were reported to be present at different levels in the FF of women with PCOS. Figure 2D provides a literature-based analysis of these lipid species in FF of women with PCOS, summarising the number of studies reporting these lipid species levels to be higher or lower compared to normo-ovulatory controls.

In the category of neutral lipids with energy storage roles, TG (52:1) was found to be consistently higher in the FF of women with PCOS across studies[63, 75]. Similarly, Prostaglandin E_2_ (PGE2), an eicosanoid derived from arachidonic acid and an important inflammatory mediator, was also found to be consistently higher in the FF of women with PCOS[78, 93]. This may be partially attributed to the higher number of recruited antral follicles in PCOS [101] that, cumulatively, increase PGE2 concentrations. Moreover, the ovarian environment in PCOS is well-documented to have a pro-inflammatory and hyperresponsive state [102], which was shown to increase PGE2 production by GCs [78].

Finally, the lipid-related antioxidant, the mitochondrial cofactor Coenzyme Q10, was also observed to display some variability across studies, with two studies reporting lower levels[67, 79], and another study reporting higher [75] levels in the FF of women with PCOS compared to normo-ovulatory controls. This finding of lower levels of Coenzyme Q10 aligns with the differences observed in water-soluble metabolites, which provide evidence of impaired mitochondrial function in PCOS. Additionally, it may also reflect the increased oxidative stress within the follicle that characterises PCOS [38].

#### Water-soluble metabolites variations

Following the same strategy as for lipids, water-soluble metabolites were organised into three categories: amino acids (Fig. 2E), energy metabolism-related metabolites (Fig. 2F), and other water-soluble metabolites (Fig. 2G), including the lipid-metabolism and nucleotide-metabolism related metabolites.

##### Amino acids

The amino acid profile in the FF of women with PCOS differed from normo-ovulatory controls (Fig. 2E). Among the branched-chain amino acids (BCAAs), isoleucine levels were consistently reported to be higher across studies [76, 97], whereas leucine and valine displayed divergent patterns. Two studies found higher leucine levels [88, 97], while another reported lower levels in the FF of women with PCOS[64]. Likewise, valine levels were reported as higher [97] in one study, but lower in another [67].

In the class of aromatic amino acids, tryptophan, phenylalanine, and tyrosine also exhibited inconsistent trends across studies. All three aromatic amino acids were reported to be at higher levels in the FF of women with PCOS in one study each (tryptophan and tyrosine [76], for phenylalanine [88]. Conversely, tyrosine [71] and phenylalanine [95] levels were reported to be lower in the FF of women with PCOS in a single study each. Tryptophan levels were found to be lower in the FF of women with PCOS compared to normo-ovulatory controls in two studies [67, 95]. 

Likewise, methionine, a sulphur-containing essential amino acid with a major role as a methyl donor for methylation processes [103], revealed inconsistent results across studies, being reported as presenting higher levels in the FF of women with PCOS in one study [76] and lower levels in another study [67].

Lysine, a basic amino acid with an important role in protein structure and epigenetic regulation [104], also showed a similarly conflicting pattern, with one study reporting higher levels [65] and another the opposite [95].

In contrast to these heterogeneous findings, threonine levels were consistently reported as being lower in the FF of women with PCOS across the reviewed studies [64, 67]. Threonine is an essential amino acid with several functions, including roles in the synthesis of non-essential amino acids and proteins, and in energy production [105]. Similarly, glutamine [76, 95], arginine [67, 71, 95], and citrulline [90, 95] levels were consistently reported as being lower in the FF of women with PCOS across the reviewed studies. Glutamine also plays several roles in the body, being particularly important for highly proliferative cells. It supports cell growth and immune function by acting as an energy source and as a precursor for the synthesis of amino acids, proteins, antioxidants, nucleotides, and neurotransmitters. Together, glutamine, citrulline, and arginine are central metabolites in the urea cycle and glutamine-citrulline-arginine axis pathways and regulate nitric oxide production and nitrogen metabolism [106].

##### Energy metabolism-related metabolites

Differences in energy metabolism-related metabolite levels were also found in the FF of women with PCOS. In particular, the intermediates of the Krebs cycle, succinate [76, 87, 97] and malate [87, 97], were found to be at higher levels in the FF of women with PCOS, possibly reflecting dysregulated mitochondrial activity. Succinate levels were reported to be lower in FF of women with PCOS in a single study [71].

In contrast, pyruvate and lactate levels, central metabolites of glycolysis and anaerobic metabolism, displayed contradictory trends, with one study showing higher levels [67, 97] and other lower levels [76, 89, 97] for both metabolites in the FF of women with PCOS. These discrepancies may reflect the heterogeneity in insulin-resistance status and glucose metabolism among women with PCOS [15, 107]. Likewise, β-Hydroxybutyrate, a ketone body, was reported to be higher [97] in one study and lower in another [64].

##### Other water-soluble metabolites

As for the lipid metabolism-related energy metabolites – glycerol and carnitine – the findings also diverged. Glycerol, the glycerolipids’ backbone, was consistently found to be at higher levels [64, 76] in the FF of women with PCOS as compared to normo-ovulatory women, and carnitine, a crucial metabolite to transport fatty acids for β-oxidation, showed inconsistent results, with one study reporting higher [65] and another lower levels in the FF of women with PCOS [71].

The nucleotide-related metabolites—hypoxanthine and uracil, associated with purine and pyrimidine metabolism, respectively, were consistently reported to be at higher levels in the FF of women with PCOS in two studies each [67, 76].

Finally, N1-Methyl-4-pyridone-3-carboxamide (4PY), the end product of the nicotinamide adenine dinucleotide (NAD) pathway, was consistently observed to be at lower levels in the FF of women with PCOS compared to normo-ovulatory controls in two studies [93, 97].

Overall, these findings highlight the widespread differences in the water-soluble metabolite profile of the FF of women with PCOS, particularly involving amino acid, energy, lipid, and nucleotide-related metabolism.

### Metabolic pathways contributing to the follicular fluid composition in PCOS

To identify which metabolic pathways are more likely to contribute to the metabolite fingerprints of the FF of women with PCOS, an enrichment analysis was conducted using all the metabolites that showed significant differences between women with PCOS and normo-ovulatory controls.

The pathway enrichment analysis results are summarised in Table 2 and Fig. 3. The results revealed several metabolic pathways likely to be involved and markedly dysregulated in PCOS. The most significantly affected pathways were the glycerophospholipid metabolism (*p* < 0.0001, FDR < 0.0001), the valine, leucine and isoleucine biosynthesis (*p* < 0.0001, FDR < 0.0001), the glycerolipid metabolism (*p* < 0.0001, FDR = 0.00219), the glyoxylate and dicarboxylate metabolism (*p* = 0.00041, FDR = 0.00592), the Krebs cycle (*p* = 0.00043, FDR = 0.00592), the biosynthesis of unsaturated fatty acids (*p* = 0.00044, FDR = 0.00592), the arginine biosynthesis (*p* = 0.00074, FDR = 0.00782), and the alanine, aspartate and glutamate metabolism (*p* = 0.00077, FDR = 0.00782). Other pathways, including sphingolipid metabolism (*p* = 0.0101, FDR = 0.0912) and steroid hormone biosynthesis (*p* = 0.0122, FDR = 0.0985), also showed significant *p-*values and FDR values < 0.1. Additional metabolic pathways, such as the arachidonic acid metabolism and the linoleic acid metabolism, the pyruvate metabolism, the valine, leucine and isoleucine degradation, the phenylalanine, tyrosine and tryptophan biosynthesis and the histidine metabolism and glycine, serine and threonine metabolism, as well as the nicotinate and nicotinamide metabolism, also showed pathway enrichment with significant *p*-values (*p* < 0.05) although with higher FDR values.

Overall, these findings highlight that shifts in lipid metabolism, steroid hormone biosynthesis, amino acid metabolism, and energy-related pathways contribute to the distinctive FF composition in PCOS.

## Discussion

This systematic review provides a comprehensive summary of the differences in the metabolite profile observed in the FF of women with PCOS compared to normo-ovulatory controls. Across the analysed studies, several consistent differences in metabolite levels in the FF of women with PCOS were identified, which belong to multiple metabolite subclasses, highlighting the molecular complexity of PCOS reflected at the follicular level.

### Androgenic-related metabolites

Analysis of the included papers revealed that PCOS is characterized by significant shifts in steroid hormone biosynthesis, leading to increased production of androgenic hormones [74, 92, 93]. These results align with the well-recognized clinical features of PCOS and the widely reported pathway shifts in ovarian steroidogenesis [25, 26, 108–115]. Hyperandrogenism is observed in approximately 75–90% of women with PCOS, and it plays a central role in the disruption of normal follicular development, contributing to ovulatory dysfunction, anovulation, or oligo-ovulation [116, 117]. In women, testosterone originates from two main sources: secretion by the ovaries and adrenal glands, and peripheral conversion of circulating precursor hormones, primarily androstenedione [118–120]. Under physiological conditions, the ovaries and the adrenal glands contribute equally to androgen production, with LH stimulating the ovaries and adrenocorticotropic hormone (ACTH) regulating the adrenal androgen synthesis. However, in PCOS, the ovary becomes the predominant source of androgen excess [120]. Androgen excess in PCOS has been hypothesised to arise from intrinsic abnormalities in theca cell function and/or dysregulation of the neuroendocrine axis. The hypothalamic-pituitary–gonadal axis serves as a central endocrine regulator of gonadal function, orchestrating the tightly coordinated processes of steroidogenesis and folliculogenesis within the ovary [121]. Steroidogenesis involves the hypothalamus’ release of GnRH in a pulsatile manner to stimulate the pituitary gland to release LH and FSH. LH acts on theca cells, stimulating the synthesis of androgens, while FSH stimulates GCs, promoting the aromatization of these androgens into estrogens, particularly estradiol [122]. In PCOS, increased pituitary sensitivity to GnRH results in heightened frequency and amplitude of LH pulses, leading to a disproportionately elevated LH:FSH ratio [23, 123–126]. This LH pulses dysregulation may result in increased production of androgens and hence increase their abundance in the FF of women with PCOS. However, the mechanisms driving persistently elevated LH pulse frequencies and amplitude in PCOS remain unclear, but several theories have been proposed over the years. The most strongly supported hypothesis proposes that hyperandrogenism itself disrupts central neural circuits responsible for steroid hormone feedback, contributing to dysregulated GnRH secretion in PCOS [127, 128]. It has also been proposed that low progesterone levels that characterize anovulation may impair the normal negative feedback on GnRH secretion, as progesterone typically decreases GnRH/LH pulsatility [129]. Another hypothesis speculates that hyperinsulinemia may directly stimulate GnRH neuron activity or enhance pituitary sensitivity to GnRH [130], although hyperinsulinemia is not invariably present in PCOS, and this hypothesis cannot serve as an explanation for those women with PCOS but without hyperinsulinemia. Nevertheless, recent literature increasingly supports that PCOS represents a primary disorder of ovarian function rather than a primary neuroendocrine defect [120]. Several studies provided evidence of an intrinsic defect in thecal cells that contributes to the hyperandrogenic state associated with PCOS. Gilling-Smith et al. provided the first direct evidence that thecal cells from polycystic ovaries exhibit elevated basal and LH-stimulated androstenedione production. Using an in vitro model, the authors also reported an increased androstenedione-to-progesterone ratio, suggesting enhanced steroidogenic conversion via 17α-hydroxylase/17,20-desmolase (encoded by CYP17A1 gene) activity [131]. Supporting these findings, Nelson VL et al. also demonstrated that theca cells from women with PCOS consistently showed an overexpression of mRNA of CYP17A1, as well as increased enzyme activity of cholesterol side chain cleavage enzyme (encoded by CYP11A1 gene), CYP17A1, 3β-Hydroxysteroid dehydrogenase type 2 (3β-HSD, encoded by HSD3B2 gene) and 17β-Hydroxysteroid dehydrogenase (17β-HSD, encoded by HSD17B1 gene) [26, 113] Interestingly, genetic association studies further support the hypothesis of an intrinsic, possibly genetically determined, abnormality of theca cells at the origin of ovarian hyperandrogenism. Recent genome-wide association studies (GWAS) have identified multiple loci associated with PCOS [132, 133]. Among the strongest candidates are the DENND1A, LHCGR, INSR, and RAB5B genes, along with adapter proteins and their downstream signalling pathways, forming a hierarchical regulatory network that converges to control androgen biosynthesis in theca cells [134–136]. Recently, McAllister et al. demonstrated the upregulation of both mRNA and protein levels of an alternatively spliced form of DENND1A (DENND1A.variant 2) in theca cells from women with PCOS. Forced overexpression of DENND1A.V2 in normal theca cells augmented CYP17A1 and CYP11A1 gene transcription, mRNA abundance, and androgen biosynthesis, whereas silencing of DENND1A.V2 in theca cells from PCOS made them revert to a normal phenotype [137]. Since then, several other studies provided evidence that strongly supports a pivotal role of DENND1A.V2 in driving ovarian hyperandrogenism associated with PCOS [138–140]. Overall, these findings consolidate the hypothesis that PCOS is a disorder of intrinsic ovarian hyperandrogenism, driven by dysregulated thecal steroidogenesis at both transcriptional and enzymatic levels. The mechanisms leading to its full-blown clinical presentation appear to be orchestrated by a combination of intrinsic genetic abnormalities, neuroendocrine perturbations, and metabolic factors.

### Progestogenic-related metabolites

**Fig. 4:**
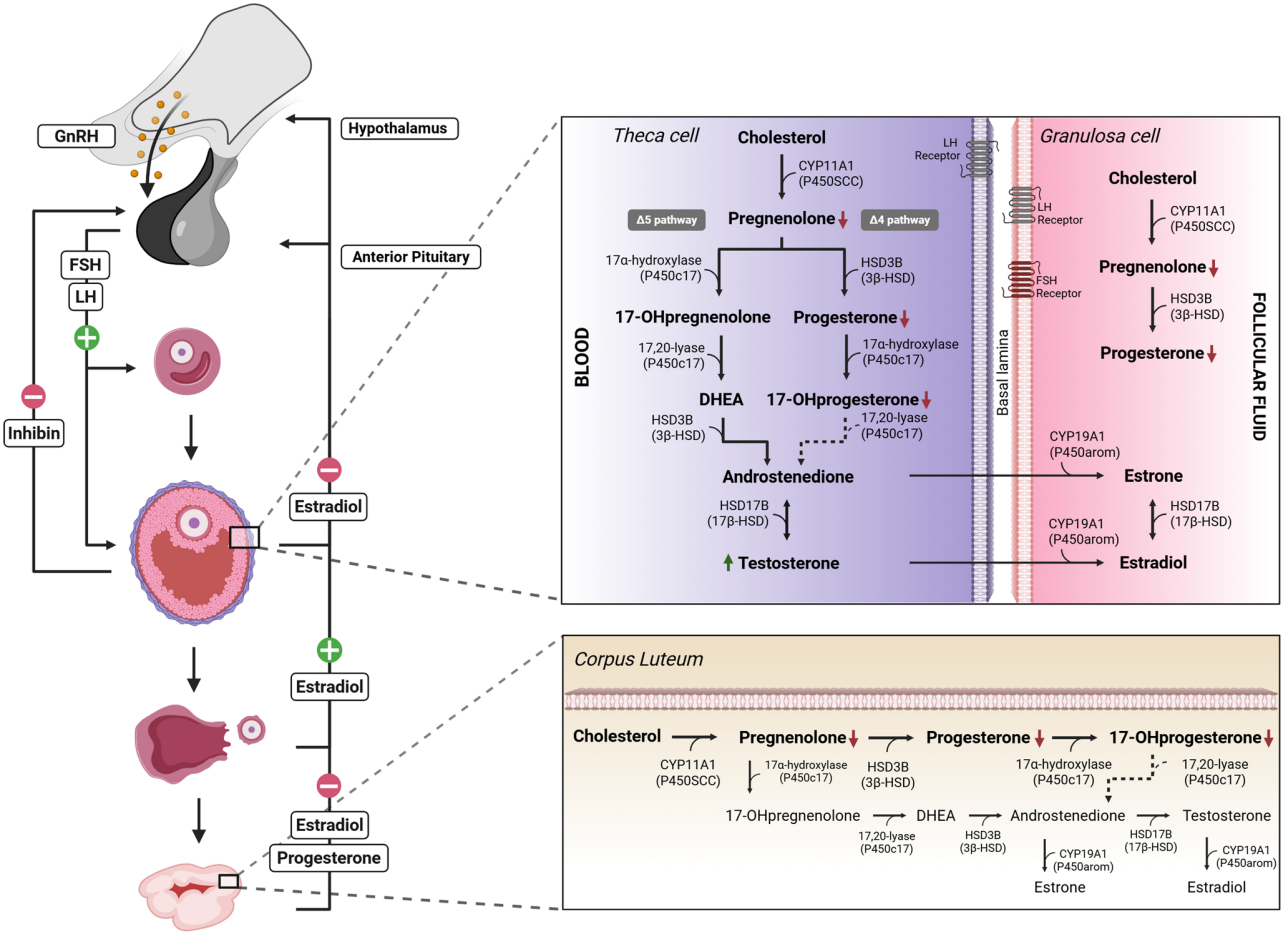
Regulation of ovarian steroidogenesis and compartment-specific steroidogenic pathways. The schematic illustrates hypothalamic–pituitary–ovarian (HPO) axis regulation of follicular development and steroid hormone production, highlighting the functional compartmentalization between theca and granulosa cells (GCs). Dashed arrows indicate limited enzymatic activity of 17,20-lyase toward 17-hydroxyprogesterone (17-OHP4)

Our analysis of the included studies indicates that PCOS is associated with profound alterations in progestogenic-related metabolites within the follicular microenvironment. The reviewed studies reported lower 17-OHprogesterone (17-OHP4) levels in the FF of women with PCOS as compared to normo-ovulatory controls [65, 93]. This intrafollicular depletion contrasts with the reports of elevated plasma 17-OHP4 in PCOS, particularly in response to gonadotropin stimulation [25, 120]. Approximately two-thirds of women with PCOS exhibit functional ovarian hyperandrogenism, a phenotype marked by exaggerated 17-OHP4 responsiveness to exogenous gonadotropins, possibly attributed to dysregulated CYP17A1 activity [120]. An ovarian follicle is organised into an avascular GC compartment closely associated with a vascularized theca cell layer, with both separated yet functionally coupled by a basement membrane. This intimate spatial arrangement reinforces bidirectional paracrine signalling and cooperative steroidogenesis, whereby theca-derived androgens diffuse to GCs for aromatisation into estrogens. Ovarian steroid biosynthesis proceeds via either the Δ4 or the Δ5 pathway. In the Δ4 pathway, pregnenolone is converted to progesterone, then to 17-OHP4 and ultimately androstenedione through the sequential action of HSD3B2 and CYP17A1 (Fig. 4). However, in humans, CYP17A1 demonstrates limited 17,20-lyase activity toward 17-OHP4; consequently, progesterone and 17-OHP4 are generally the terminal products of the Δ4 pathway in theca cells [141, 142]. Since CYP17A1 is exclusively expressed in theca cells, 17-OHP4 in FF is presumably derived from those cells. Recent studies have elucidated the dynamic steroidogenic interplay between theca and GCs during the late follicular phase [142, 143]. While FF progesterone levels rise dramatically, the 17-OHP4 levels in FF show only a slight increase. In contrast, serum 17-OHP4 rises significantly during this period, indicating that theca cell-derived 17-OHP4 is mainly secreted into the systemic circulation. This is likely due to the highly vascularized nature of the theca layer, in contrast to the avascular granulosa layer [142]. These patterns highlight distinct intraovarian versus systemic dynamics regarding steroidogenic regulation. Notably, although 17-OHP4 is a poor substrate for 17,20-lyase activity in isolated theca cells, conversion of 17-OHP4 to androstenedione has been documented in ovarian and adrenal tissue fragments [144]. These observations are compatible with the possibility that paracrine interactions within the follicular microenvironment, potentially involving GCs–derived factors, may enhance 17,20-lyase activity toward this substrate, or that additional enzymatic pathways contribute to this conversion in vivo [120, 145]. Moreover, a contribution of adrenal steroidogenesis to the circulating levels of 17-OHP4 cannot be excluded. A subset of women with PCOS was shown to depict increased secretion of adrenocortical steroids, both in unstimulated and ACTH-stimulated conditions, which may contribute to circulating 17-OHP4 levels without directly impacting the follicular microenvironment [146]. Such an adrenal contribution would be expected to preferentially influence systemic hormone levels rather than intrafollicular concentrations, thereby further accentuating the observed dissociation between blood and FF steroid profiles in PCOS.

The reviewed studies also consistently demonstrated lower concentrations of both pregnenolone and progesterone in the FF of women with PCOS [71, 76, 82, 92–94, 147], although pregnenolone levels show some inconsistencies [94]. Among the five studies that examined FF pregnenolone, only Yu L et al. reported higher levels in PCOS; however, the small sample size of the study limits the generalizability of this finding [94]. Moreover, these discrepancies are likely to reflect the heterogeneity of PCOS phenotypes and methodological differences across metabolomic platforms. Under physiological conditions, pregnenolone is synthesised from cholesterol by CYP11A1 in both theca and GCs and serves as the universal precursor for all ovarian steroids. Particularly in the Δ4 pathway, pregnenolone is irreversibly converted to progesterone. Progesterone is a dominant steroid in the preovulatory follicle after the LH surge and plays a pivotal role in ovulation, embryo development, and maintenance of pregnancy [148]. Although both theca and GCs can produce progesterone, GCs assume primary responsibility for progesterone synthesis in the late follicular maturation [142, 143]. Marie C., et al. reported marked downregulation of CYP11A1 and HSD3B2 expression in GCs from women with PCOS, providing a molecular basis for diminished pregnenolone and progesterone biosynthesis [82]. Further, reduced expression of the steroidogenic acute regulatory protein (StAR), a key facilitator of cholesterol transport into mitochondria, has been implicated in impaired progesterone production in PCOS GCs [44, 149]. PCOS is not only characterized by hyperandrogenism but also by GCs-mediated progestogenic insufficiency. Defective cholesterol transport and downregulated steroidogenic enzymes impair the steroidogenic pathways, leading to lower levels of pregnenolone and progesterone in the follicular microenvironment. Collectively, these findings support the hypothesis that GCs dysfunction could drive the intrafollicular deficiency of progestogenic metabolites that also characterise PCOS. This progestogenic imbalance likely contributes to follicular arrest, anovulation, and luteal phase defects in PCOS.

### Estrogenic-related metabolites

Our review highlights inconsistent findings regarding estrogen levels, particularly estradiol, in the FF of women with PCOS across different studies [82, 90, 94]. Dysregulated steroidogenesis in PCOS extends beyond theca cell hyperactivity and impacts GCs function, contributing to follicular arrest. Androstenedione produced by theca cells diffuses across the basement membrane into GCs, where it is converted into estradiol by the enzyme cytochrome P450 family 19 subfamily A member 1 (encoded by CYP19A1 gene) under the action of FSH (Fig. 4). Marie C. et al. showed that overweight, non‑hyperandrogenic women with PCOS had lower FF estradiol levels, which correlated with downregulation of CYP11A1 and HSD3B2 expression in GCs [82]. The authors hypothesized that these lower estradiol concentrations may reflect impaired estradiol signalling rather than a primary defect in estradiol biosynthesis [82]. In contrast, Yu L. et al. found higher levels of estriol and estradiol in the FF of women with PCOS. Notably, this study also found mRNA overexpression of key steroidogenic enzymes (CYP11A1, CYP19A1, and HSD17B2) in FF-derived exosomes, suggesting altered estrogen biosynthesis in women with PCOS [150]. A similar finding was reported by Xu Y. and colleagues, who also found higher levels of estradiol in the FF of women with PCOS. Overall, there is no consistency in the data concerning estradiol levels in the FF of women with PCOS. Notably, studies reporting elevated FF estradiol levels were conducted in relatively small cohorts and included women with PCOS who were normal-weight and exhibited hyperandrogenism, whereas the study by Marie C. et al., which reported reduced FF estradiol concentrations, focused on women with PCOS who were overweight and non-hyperandrogenic. These differences in population size, BMI and androgenic phenotype could partially account for the observed discrepancies, highlighting the influence of body weight and androgens on intrafollicular estrogen signalling in PCOS. Several studies have shown that GCs from women with PCOS often display decreased CYP19A1 expression and activity, impairing the aromatization of androgens into estrogens. A study involving women with PCOS and metabolic syndrome found that increased expression of C-terminal binding protein 1 (CTBP1) in GCs represses CYP19A1 transcription by directly binding to promoter II, thereby reducing estradiol synthesis [150]. Moreover, exposing GCs to testosterone levels similar to those observed in the FF of women with PCOS, downregulates aromatase, suggesting that hyperandrogenism itself impairs estrogen production [151]. Another proposed mechanism involves increased 5α-reductase activity in GCs, leading to elevated levels of 5α-androstane-3,17-dione, a potent endogenous aromatase inhibitor. The accumulation of this metabolite further suppresses estrogen biosynthesis and exacerbates androgen dominance within the follicle [111, 152, 153]. Notably, estradiol levels in FF of women with PCOS are highly variable, reflecting a dynamic interplay between hyperandrogenism and GCs dysfunction. This pattern of abnormalities converges to impair estrogen production, contributing to follicular arrest and anovulation in PCOS. Future research should stratify women by phenotype and employ standardized assays to clarify the determinants of the pattern of estradiol in the FF of women with PCOS and their implications for folliculogenesis.

### Free fatty acids

Metabolomic analyses consistently reveal that women with PCOS exhibit broad differences in FF FFAs composition, encompassing higher levels of SFAs, MUFAs, and PUFAs (Fig. 2B). These intrafollicular observations are in line with those reported for circulating levels [154, 155], supporting the hypothesis that the FF composition is strongly influenced by systemic metabolism. Given that the FF composition is, at least in part, derived from plasma through transudation across the blood-follicle barrier, changes in fatty acid profiles in the FF of PCOS may partially reflect its circulating levels. Notwithstanding, because FFA and other complex molecules are transported in circulation bound to lipoproteins rather than being freely diffusible, their levels in the FF also reflect their selective transport across the blood-follicular barrier, uptake, metabolism, and remodelling by granulosa and theca cells, instead of simply mirroring plasma availability [156]. Notably, our pathway enrichment analysis pointed to the biosynthesis of unsaturated fatty acids, the linoleic acid metabolism, the alpha-linolenic acid metabolism, the arachidonic acid metabolism, the fatty acid biosynthesis, the fatty acid elongation, and the fatty acid degradation as metabolic pathways potentially impaired within the follicles of women with PCOS (Fig. 3).

Among the SFAs identified at higher levels in the FF of women with PCOS –including C14:0, C16:0, C18:0, and C22:0 – the most consistent results were observed for C16:0 and C18:0 [73, 77, 81, 83, 90]. Among these, C16:0 levels were found to be significantly higher in the FF across multiple PCOS phenotypes. In fact, Ding et al. conducted subgroup analyses and showed that C16:0 levels in the FF were more pronounced in women with PCOS who also had obesity, insulin resistance, and hyperandrogenism as compared to women with PCOS but without these features [73]. Moreover, C16:0 was shown to be excellent at discriminating between women with PCOS and insulin resistance from those without it, with an area under the curve (AUC) of 0.827 on ROC curve analysis for distinguishing between women with PCOS with and without insulin resistance, indicating that C16:0 could be used as a very good discriminant of metabolic phenotypes of PCOS [73]. Supporting these results, a metabolomic profiling study of the FF of normal-weight women with PCOS showed that those with insulin-resistance had significantly higher levels of C16:0 and C18:0 compared with normal-weight women with PCOS without insulin-resistance [157]. Insulin is crucial to regulate lipid metabolism by suppressing adipocyte lipolysis via inhibition of hormone-sensitive lipase in adipocytes; however, under conditions of insulin resistance, the anti-lipolytic effect is impaired, leading to increased adipose tissue lipolysis and elevated circulating FFAs [158]. The resulting excess FFAs are taken up and accumulate in non-adipose tissues, including the ovaries, where they exceed the cells’ metabolic capacity to oxidize them through β-oxidation or safe storage as TGs [159]. This leads to the intracellular accumulation of FFA-derived lipids – acyl-CoAs, ceramides, and DAGs – which disrupts mitochondrial and endoplasmic reticulum function, promotes oxidative stress and inflammation [160], and interferes with signalling pathways, such as PI3K-Akt and PKC pathways, contributing to the lipotoxic impairment of insulin signalling and metabolic dysfunction [159–162]. Xu Y. et al., also found C16:0 and C18:0 levels to be higher in the FF of women with PCOS, and particularly higher levels in those women with hyperandrogenism [90]. Interestingly, in ovarian GCs in vitro*,* both C16:0 and C18:0 were demonstrated to induce apoptosis through the metabolism of the respective acyl-CoA forms [163], while C16:0 was shown to impair glucose metabolism by interfering with the classical PI3-K/Akt pathway by activating the JNK pathway [164]. Thus, this lipotoxic state might be one of the mechanisms through which insulin resistance adversely affects follicular development and quality, which deserves further investigation to understand both the cause and the consequences of increased FFAs, and in particular of C16:0 and C18:0, in the follicular microenvironment in PCOS.

The fact that C16:0 levels were also significantly higher in the FF of women with PCOS and obesity when compared to their normal weight counterparts reinforces C16:0 accumulation as an obesity-associated metabolic signature [73, 83]. Importantly, this differential enrichment suggests that C16:0 may serve as a potential discriminator of PCOS phenotypes, thereby providing valuable insight into phenotype-specific metabolic disturbances and contributing to a deeper understanding of PCOS mechanisms.

The studies included in this review also reported elevated MUFAs levels in the FF of women with PCOS, including C14:1, C16:1, and C18:1, among which the most consistent results were found for C16:1 and C18:1 [73, 77, 81, 83, 87, 90]. In the study conducted by Ding and colleagues, both C16:1 and C18:1 levels were found to be elevated in the FF of women with PCOS [73]. Additionally, Xu. Y et al., reported C18:1 levels to be higher in the FF of women with PCOS and hyperandrogenism as compared to both women with PCOS but without hyperandrogenism and healthy normo-ovulatory controls [90]. Ding et al. found similar results, with C18:1 being particularly higher in the FF of women with PCOS and hyperandrogenism or insulin resistance. ROC curve analysis further demonstrated that C18:1 presented the highest AUC (0.903) for discriminating between PCOS phenotypes according to hyperandrogenism [73]. Moreover, elevated levels of C18:1 have been associated with obesity, as women with PCOS and obesity were described to display higher levels both in FF and plasma when compared to women with PCOS without obesity and healthy normo-ovulatory controls [83]. Notably, C18:1 was shown to upregulate pro-inflammatory cytokine expression in GCs in vitro*,* by activating the ERK1/2 signalling pathway, indicating a possible mechanistic link between the FF lipid profile and ovarian inflammation in PCOS [77]. Sun Y. et al. found that C16:1 levels in the FF were associated with increased risk of ovarian hyperstimulation syndrome (OHSS) both in normo-ovulatory and women with PCOS (adjusted odds ratio of 4.215 (95%CI 1.566–11.344), *p* = 0.004). Its elevation was accompanied by a higher C16:0/C16:1 ratio, suggestive of an increased activity of stearoyl-CoA desaturases (SCD) [87]. Elevated SCD activity may therefore contribute to the distinctive lipid milieu that predisposes women to the exaggerated ovarian response characteristic of OHSS [87].

**Fig. 5:**
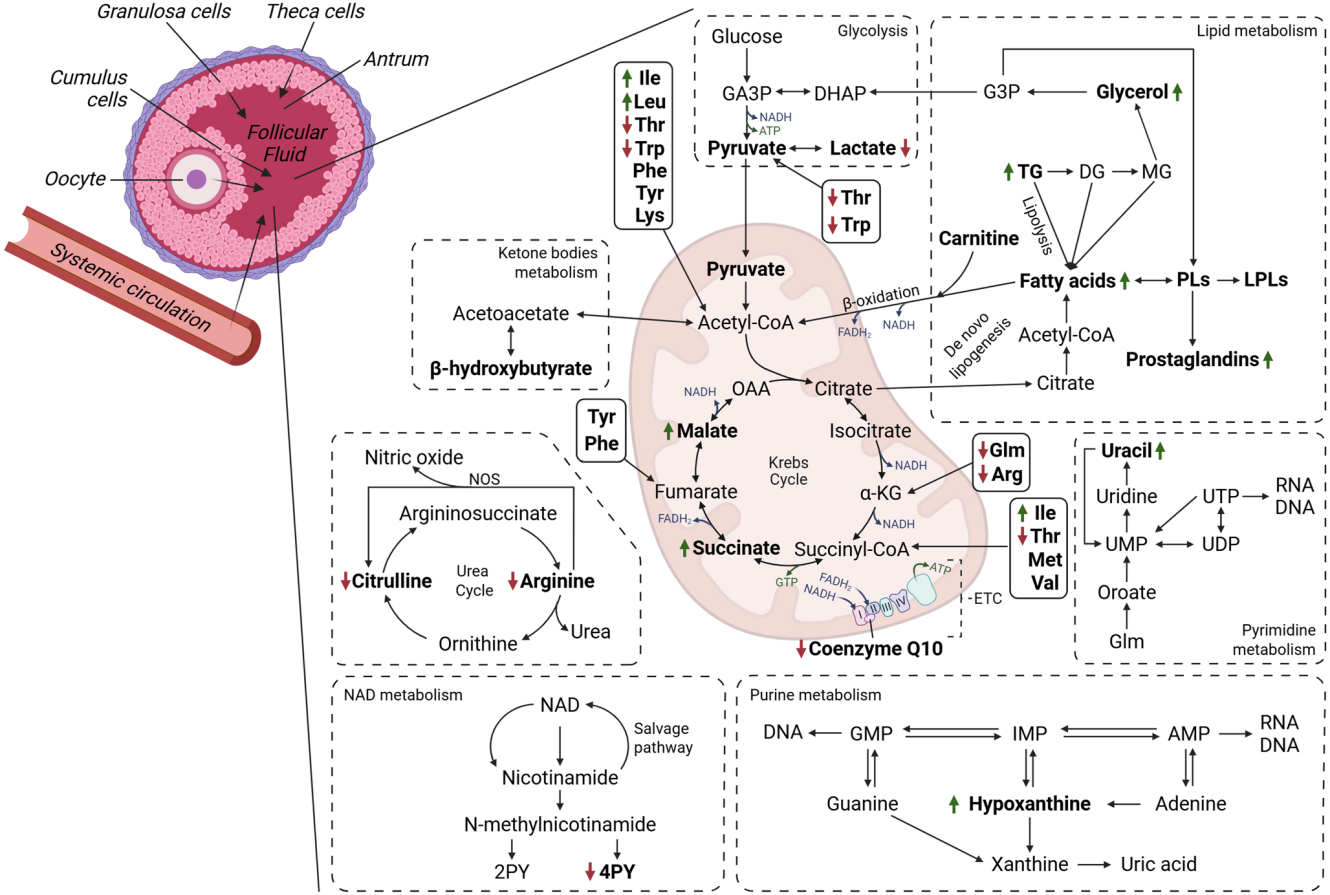
Schematic representation of metabolic alterations found through follicular fluid (FF) metabolomics as being associated with PCOS. The figure integrates metabolomic changes consistently reported in the FF of women with PCOS and illustrates how these alterations likely reflect intracellular metabolic processes occurring predominantly in granulosa and theca cells, with additional contributions from cumulus cells, the oocyte, and systemic metabolic inputs via plasma transudate. The metabolic pathways shown should therefore be interpreted as cellular processes whose intermediates and products are detectable in the FF, rather than as reactions occurring directly within the FF itself. Metabolites shown in bold correspond to those reported as significantly altered in the FF of women with PCOS compared to normo-ovulatory control women in at least two independent studies. Metabolites depicted in normal font represent key intermediates included to provide pathway continuity and biological context, but were not consistently reported as altered in the included studies. Up (↑)- and down (↓) -arrows indicate the predominant direction of change reported in the FF of women with PCOS relative to controls, with green up-arrows denoting increased levels and red down-arrows denoting decreased levels in the FF of women with PCOS. Bold metabolites without up- or down-facing arrows indicate inconsistent findings across studies, with similar numbers of articles reporting increases and decreases, precluding a clear directional trend. Black arrows indicate established biochemical metabolic relationships and do not imply PCOS‑specific causal mechanisms. The FF of women with PCOS exhibits marked alterations in lipid metabolism, including increased fatty acids, phospholipids (PLs), lysophospholipids (LPLs), triglycerides (TG), and prostaglandins, consistent with disrupted lipid homeostasis and a pro-inflammatory environment. Central carbon metabolism is perturbed, with alterations in glycolysis and the tricarboxylic acid (Krebs) cycle, alongside accumulation of intermediates such as malate and succinate, suggestive of mitochondrial dysfunction. Amino acid metabolism is dysregulated, characterized by increased branched-chain amino acids and reduced threonine, while purine and pyrimidine metabolism are also altered, with elevated hypoxanthine and uracil, reflecting changes in nucleotide turnover. Reduced coenzyme Q10 and altered NAD⁺-dependent processes further support impaired oxidative phosphorylation and redox imbalance. Collectively, these metabolic disturbances highlight disrupted energy metabolism, lipid homeostasis, and metabolic–inflammatory crosstalk within the ovarian follicle, which may contribute to impaired GCs function, compromised oocyte competence, and follicular arrest in PCOS. Abbreviations: 2PY – N1-methyl-2-pyridone-5-carboxamide; 4PY – N1-methyl-4- pyridone-3-carboxamide; α-KG – alpha-ketoglutarate; AMP – Adenosine monophosphate; Arg – Arginine; ATP – Adenosine triphosphate; DG – Diacylglycerol; DHAP – Dihydroxyacetone phosphate; ETC – Electron transport chain; FADH2 – Reduced flavin adenine dinucleotide; GA3P – Glyceraldehyde-3-phosphate; G3P – Glycerol-3-phosphate; Glm – Glutamine; GMP – Guanosine monophosphate; Ile – Isoleucine; IMP – Inosine monophosphate; Leu – Leucine; Lys – Lysine; Met – Methionine; MG – Monoacylglycerol; NAD – Nicotinamide adenine dinucleotide; NADH – Reduced nicotinamide adenine dinucleotide; NOS – Nitric oxide synthase; OAA – Oxaloacetate; PCOS – Polycystic ovary syndrome; Phe – Phenylalanine; TG – Triacylglycerol; Thr – Threonine; Trp – Tryptophan; Tyr – Tyrosine; UDP – Uridine diphosphate; UMP – Uridine monophosphate; UTP – Uridine triphosphate; Val – Valine

Among the PUFAs identified as being elevated in FF of women with PCOS – including C18:2, C18:3, C20:4, C20:5, and C22:5 – the majority of studies report higher levels of C18:2 and C20:4 [73, 77, 81, 87, 90]. Ding et al. showed that among PCOS phenotypes, those with insulin-resistance exhibited the most pronounced C20:4 accumulation within the FF. Curiously, elevated C20:4 levels have been shown to impair GC function by reducing total antioxidant capacity, increasing reactive oxygen species (ROS) production, and promoting mitochondrial dysfunction [81]. C20:4 serves as a substrate for cyclooxygenase (COX)‐mediated synthesis of prostaglandins (Fig. 5). Within the ovary, prostaglandin E₂ (PGE₂) is a critical regulator of ovulatory processes, including meiotic maturation, cumulus expansion, and follicle rupture [165–167]. Elevated C20:4 levels in the FF could lead to excessive prostaglandin production and local inflammation, which in turn affects the follicle development and ovulation. This hypothesis is supported by the fact that two studies included in this review reported higher levels of PGE_2_ in FF of women with PCOS [78, 93]. Li et al. reported that the FF of women with PCOS without obesity had markedly higher levels of C20:4-derived metabolites generated by COX-2 and cytochrome P450 (epoxygenase) pathways [78]. Moreover, COX–2–derived prostaglandins in the FF were shown to be positively correlated with serum testosterone and fasting insulin levels [78]. Indeed, insulin (but not testosterone) was demonstrated to strongly amplify COX-2 expression and PGE₂ secretion in human GCs *in* *vitro* [78]. Together, these findings suggest the existence of an interplay between metabolic and inflammatory cascades, wherein hyperinsulinemia promotes local inflammation and oxidative stress, ultimately contributing to impaired folliculogenesis and anovulation that characterizes PCOS. Furthermore, elevated levels of C18:2 in the FF were found to be associated with overweight status and hyperandrogenism [81, 90]. More recently, the putative roles of C18:2 and C18:3 in PCOS-related ovarian dysfunction have also been elucidated. Zhang, W. et al. demonstrated that in human GCs, C18:2 binds to the estrogen receptor to activate FOXO1 transcription, triggering downstream ROS-mediated oxidative stress and NFκB–driven inflammation, which culminates in apoptosis [168] thereby supporting the role of C18:2 in triggering GCs apoptosis—a critical driver of follicular atresia. C18:3 has also been linked to the low-grade chronic inflammation characteristic of PCOS, through upregulation of the expression and secretion of pro-inflammatory cytokines [169]. Elevated C18:3 FF levels have been observed in the FF of women with PCOS, and particularly in those with insulin resistance and hyperandrogenism [73, 90]. Additionally, Xu et al. identified C18:3 as the PUFA in FF of women with PCOS with the highest variable importance in projection score. Besides, C18:3 has been shown to directly impair oocyte maturation by increasing the incidence of meiotic defects, including spindle disorganisation and chromosome misalignment, in fully grown germinal vesicle oocytes cultured in vitro [90].

Overall, the data suggest that the lipidome of the FF of women with PCOS exhibits a lipotoxic signature characterised by the accumulation of SFAs, MUFAs, and PUFAs that collectively drive oxidative stress, inflammation, and GCs dysfunction. Phenotype‐specific elevations, most pronounced in women with obesity, insulin resistance, and hyperandrogenism, underscore the interplay between systemic metabolic profile and local follicular microenvironment.

### Phospholipids

Phospholipids are a type of lipid molecule that forms the basic structure of all cell membranes. Several phosphatidylethanolamine (PE) species, a subclass of phospholipid that appears in biological membranes, particularly in the inner portion of the cell membrane and in mitochondrial membranes, were consistently found to be higher in the FF of women with PCOS – including PE(34:1), PE(34:2), PE(36:2), PE(36:4), PE(38:5), PE(38:6) and PE(40:6) – with higher levels of PE(34:2) and PE(36:2) consistently reported across multiple studies [63, 84, 93]. PE is a major phospholipid in cell membranes that influences membrane curvature and fluidity [170]. Unbalance between lipid classes, with relatively lower PE levels, has been linked to lipotoxic stress in the pathogenesis of multiple disorders, including metabolic and neurodegenerative diseases [171, 172]. Qian et al. demonstrated that PE levels in the FF were positively correlated with serum total testosterone and AMH concentrations, while negatively correlated with embryo quality. These findings suggest that PE accumulation may reflect both the severity of PCOS and serve as a prognostic biomarker of adverse reproductive outcomes [84]. Specifically, PE(34:2) and PE(36:2) were identified among the top metabolites discriminating women with PCOS from normo-ovulatory controls in untargeted metabolomic analyses [93]. In line with these observations, a recent study among 179 plasma lipid species, identified high levels of PE(34:2) and PE(36:2) as putative risk factors for PCOS [173]. Notwithstanding, the current literature is limited, and the underlying mechanisms remain unclear. Further studies are essential to clarify the role of PE in PCOS.

The reviewed studies revealed inconsistent findings on phosphatidylcholine (PC) species in the FF of women with PCOS [72, 75, 96]. PC, a major phospholipid in cell membranes, plays a key role in maintaining cellular structure and function. PC has been shown to support cell proliferation and programmed cell death, and enhance insulin sensitivity [174]. While PC (36:3) levels were consistently found to be lower, findings on PC (36:4) levels were inconsistent, with studies reporting either higher or lower levels in the FF of women with PCOS as compared to normo-ovulatory controls. Still, low PC levels in the FF of women with PCOS were positively correlated with LH/FSH [79]. In contrast, Mendelian randomization using GWAS data indicates that higher circulating levels of PC are associated with increased risk of PCOS, suggesting that PC excess may also contribute to the condition [175]. Additionally, elevated PC levels in the FF were also associated with reduced embryo quality on day 3 post-fertilisation [176]. The relationship between PC levels in the FF of women with PCOS appears to be complex, with studies reporting both lower and higher levels. The available literature remains too limited to explain these discrepancies or to support robust mechanistic hypotheses. Further research is needed to elucidate the role of PC in PCOS, as well as its impact on fertility.

### Lysophospholipids

Lysophospholipids are a class of membrane lipids that are derived from phospholipids after the removal of one fatty acid chain, by the action of phospholipase enzymes. Reported levels of lysophospholipids in the FF of women with PCOS, compared with normo-ovulatory controls, were inconsistent across studies. Lysophosphatidylcholine (LPC) and lysophosphatidylethanolamine (LPE) are lipids involved in various signalling pathways. For instance, LPC seems to modulate glucose uptake and insulin signalling [177, 178], whereas LPE appears to influence lipid accumulation by modulating the genes involved in lipogenesis and lipolysis [179]. LPC(16:0) was found to be lower in the FF of women with PCOS, as reported by two studies [85, 88], whereas another study reported the opposite [79]. Circulating levels of LPC(16:0) were also found to be lower in women with PCOS and obesity or normal weight when compared to control women with similar corpulence [180]. A similar trend was observed for LPC(18:2), which was reported to be lower in the FF of women with PCOS in two studies [84, 85], but higher in another [79]. Overall, evidence regarding LPC species levels in PCOS is very scarce, and the results are conflicting. This heterogeneity may reflect differences in PCOS phenotypes or the population characteristics, such as BMI. In contrast, LPE(16:0) levels tend to be higher in the FF of women with PCOS compared to normo-ovulatory controls, as reported by two studies [79, 93], whereas another reported lower levels [74]. Evidence for LPE in PCOS is scarce, but the differences observed across studies imply significant shifts in the profile of glycerophospholipids within the follicular microenvironment in PCOS, as evidenced in our over-representation analysis (Fig. 3).

Notably, lysophosphatidic acid (LPA)(18:1) has been reported to be present at lower levels in the FF of women with PCOS [74, 85]. LPA is a small bioactive lipid that exerts potent extracellular signalling through its interaction with its six specific G protein-coupled receptors (GPCRs), mediating important responses, such as cell proliferation, migration, and cytoskeletal reorganisation [181, 182]. Within the ovarian follicle, LPA receptor mRNA has been detected in granulosa–lutein cells from women undergoing IVF, supporting a functional role for LPA signalling within the follicular milieu [183]. Mechanistic studies further show that LPA(18:1) modulates AKT activity in a GCs line (KGN cells) and that enhanced LPA(18:1) signalling activates the PI3K–AKT–mTOR pathway via LPAR1 in GCs, thereby suppressing apoptosis through induction of autophagy [184]. Consistent with these observations, LPA(18:1) supplementation of maturation medium improves bovine oocyte maturation rates in vitro, reduces apoptosis in cumulus–oocyte complexes, and sustains expression of factors linked to developmental competence [185]. Collectively, these findings link LPA signalling to GCs survival and oocyte maturation; therefore, reduced LPA availability in the follicular microenvironment could plausibly contribute to impaired follicular development and poorer oocyte quality in PCOS. Nevertheless, well-designed studies integrating lipidomic profiling with functional analyses are needed to define the role of LPA signalling in PCOS.

Overall, the shifts in LPC, LPE and LPA levels suggest a disruption of lipid signalling and metabolism within the follicular environment in PCOS, which may contribute to impaired follicular function and oocyte quality. However, further studies dedicated to unravelling the molecular mechanisms regulating glycerophospholipid metabolism and their physiological roles within the follicular environment are needed to clarify whether these act as culprits or bystanders in PCOS.

### Triglycerides

Triglyceride (TG) (52:1), consisting of a glycerol backbone bound to three fatty acids C16:0, C18:0, and C18:1, was reported to be significantly higher in the FF of women with PCOS compared to normo-ovulatory controls in two studies [63, 75]. Likewise, TG(52:1) levels were found to be higher in the serum of women with PCOS [186], suggesting that the FF levels are aligned with the systemic profile rather than being an ovarian-specific feature. Besides TG(52:1), several other TG species and other lipids that serve an energetic reserve role, such as diacylglycerols (DGs), were found to be at higher levels in the FF of women with PCOS [63, 75, 84]. Ban Y et al. also reported that total TG levels were significantly higher in the FF of women with PCOS compared to normo-ovulatory controls, and these results were found to be independent of obesity and hyperandrogenism, since the mean BMIs were both within the normal range, and testosterone levels did not differ between groups [63]. Another study reported an association between higher serum TG levels and IR, marked by a positive correlation between the triglyceride-glucose (TyG) index (reflecting fasting TG and glucose) and HOMA-IR [187]. Additionally, Hestiantoro A. et al. found that TG levels were positively correlated with free testosterone index and were determinant of hyperandrogenism in women with PCOS [188], suggesting that elevated TGs in PCOS could be associated with hyperandrogenism and IR. From a systemic perspective it is well-recognized that insulin resistance is associated with reduced lipoprotein lipase activity in adipose tissue and increased release of FFAs, leading to an excess lipid flux to the liver and enhanced hepatic lipid synthesis [189, 190]. However, to the best of our knowledge, no studies have explored the mechanism underlying high TG levels in the intrafollicular environment; nevertheless, some hypotheses can be proposed. From this systematic review, we concluded that women with PCOS have significantly higher levels of FFAs in the FF, including higher levels of C16:0, C18:0, and C18:1. Since the FF results, at least in part, from the active transport of plasma components into the follicular antrum, combined with local secretions, primarily from GCs, the FFAs taken up by the ovarian cells are likely to reflect the systemic abundance, followed by excess FFAs esterification into TGs [156]. Another hypothesis that does not exclude the previous one could be impaired lipolysis within the follicle, resulting in the accumulation of energy storage lipids. Altogether, these findings suggest that the accumulation of TG(52:1) and other TGs in the FF of women with PCOS may result from systemic metabolic disturbances and altered lipid handling within the follicle. Excess TGs can lead to lipid overload in GCs and oocytes, where their hydrolysis releases free fatty acids beyond the cells’ metabolic or storage capacity, potentially triggering oxidative stress and mitochondrial dysfunction [161, 191]. This process could further contribute to generating a lipotoxic microenvironment that disrupts cellular signalling, energy homeostasis, and potentially impairs oocyte development, maturation, and ultimately impacts fertility [192, 193]. Also, the predominance of saturated fatty acids in this TG species may exacerbate these deleterious effects, further compromising follicular function and oocyte quality [191]. Nevertheless, further studies are needed to elucidate the mechanisms underlying TG accumulation in the ovarian microenvironment of PCOS, as well as its impact on oocyte quality.

### Amino acids

Among the amino acids found to be significantly altered in the FF of women with PCOS are the essential BCAAs isoleucine, leucine, and valine. These amino acids are known to play key roles in muscle metabolism, energy production, and cellular signalling. Specifically, leucine activates the mTOR pathway to stimulate protein synthesis, isoleucine enhances glucose uptake and supports haemoglobin synthesis, and valine contributes to muscle repair and nitrogen balance [194]. Among these, isoleucine and leucine levels were found to be higher in the FF of women with PCOS [76, 88, 97], although the findings on leucine were inconsistent across the studies that reported significant differences [64]. While for valine, the results were inconsistent, with one study reporting higher levels in the FF of women with PCOS [97], whereas another reported the contrary [67]. Zhao H. et al. also observed that valine and leucine levels were significantly higher in women with PCOS and obesity than in those with PCOS without obesity [97]. Consistent with these results, Sun Z. et al. found higher levels of leucine in the FF of women with PCOS [88]. In contrast, Castiglione Morelli MA. et al. observed lower leucine levels, and Lazzarino G. et al. reported lower valine levels in the FF of women with PCOS [64, 67]. These discrepancies may be attributed to potential phenotypic differences in study populations, as well as their heterogeneity and the small population size of the study by Castiglione Morelli MA. et al. [64]. In fact, while Zhao H. et al. [97] describe the phenotype of women with PCOS as having a significantly higher BMI, androgen levels, and HOMA-IR compared to normo-ovulatory controls, in Sun Z. et al. [88], Castiglione Morelli MA. et al. [64], and Lazzarino G. et al. [67] studies, no information on the phenotype of the women with PCOS, particularly for HOMA-IR, is provided. In plasma, circulating levels of BCAAs, including leucine and valine, are well-established hallmarks of insulin resistance [195]. Plasma levels of BCAAs were also found to be elevated in women with PCOS compared to normo-ovulatory controls [196, 197]. Additionally, Paczkowska K. et al., reported circulating levels of total BCAAs, leucine, isoleucine, and valine to be significantly higher in women with PCOS and IR as compared to normo-ovulatory controls, whereas in the subgroup of women with PCOS but without insulin-resistance, only isoleucine levels were significantly higher [198]. Hyperandrogenism is also a possible reason for discrepancies, since significantly higher circulating levels of isoleucine and leucine were reported in women with PCOS and hyperandrogenism, as compared to women with PCOS without hyperandrogenism [198]. The same study also found plasma BCAAs levels to be positively correlated with BMI, HOMA-IR, waist circumference, total testosterone levels, free androgen index (FAI) and percentage of fat mass, and negatively correlated with estradiol, percentage of muscle and fat-free body mass [198]. Similarly, Chang AY. et al. reported a positive correlation between plasma BCAAs and HOMA-IR and free testosterone, and a negative correlation with insulin sensitivity and SHBG [196]. Thus, it is plausible to hypothesize that the population of the study by Sun Z. et al. [88] might be IR, and the two other study populations were more likely insulin sensitive, explaining the discrepancies observed for leucine and valine. Despite these inconsistencies, a growing body of evidence highlights the association of BCAAs metabolism with PCOS. In fact, a recent study explored the link between BCAAs metabolism and the pathogenesis of PCOS, particularly involving *PPM1K,* a gene that regulates BCAAs catabolism [199]. In this work, BCAAs levels were found to be significantly elevated both in plasma and FF of women with PCOS, and FF levels of the three BCAAs were found to be positively correlated with testosterone levels and menstrual cycle intervals. Also, using both an *in silico* and an *in vivo* approach, the authors identified the *PPM1K* as a potential contributor to the metabolic and hormone alterations in PCOS. The study found that female mice lacking Ppm1k had elevated plasma BCAA levels and developed PCOS-like features, including hyperandrogenism and abnormal follicle development. Moreover, the endocrine and ovarian dysfunction in these mice was significantly improved by decreasing BCAAs dietary intake [199]. In human GCs, the knockdown of *PPM1K* shifted cellular metabolism from glycolysis to the pentose phosphate pathway and suppressed mitochondrial oxidative phosphorylation, suggesting a potential direct impact on energy metabolism within ovarian cells [199]. In our analysis, both valine, leucine and isoleucine biosynthesis and degradation were identified as enriched pathways, further supporting the association of BCAAs’ with PCOS (Fig. 3). Furthermore, BCAAs levels in the FF of women with PCOS are aligned with the majority of the results observed for these amino acids in the blood [196–198], which suggests that the metabolic shifts leading to BCAAs levels found at the FF may reflect, at least partially, a systemic rather than a local event of the follicle. Overall, these findings collectively support the link between altered BCAAs’ metabolism and the metabolic and endocrine disturbances observed in PCOS, highlighting both the potential role of BCAAs as biomarkers of IR in PCOS and the need for further research into the mechanisms by which isoleucine and potentially leucine are elevated in the FF, as well as its implications for the follicular development and oocyte quality.

In contrast, threonine consistently exhibited lower levels in the FF of women with PCOS compared to normo-ovulatory women across the studies included in this review [64, 67]. Intriguingly, discrepant results were observed for threonine levels in blood and FF of women with PCOS, as most studies report elevated threonine levels in the blood of women with PCOS compared to control women [197, 200, 201], suggesting a specific shift in the metabolism of this amino acid within the follicle. This hypothesis was previously suggested by Zhang CM. et al., who acknowledged the differences in systemic and local amino acid profiles in women with PCOS [202]. To the best of our knowledge, no hypotheses have been proposed for the lower threonine levels found in the FF of women with PCOS. Threonine is an essential amino acid that plays a vital role in the modulation of nutritional metabolism, in other amino acids and nucleotide synthesis, redox homeostasis, and epigenetic regulation, all of which are vital for oocyte development. Additionally, threonine can be metabolised into several Krebs Cycle intermediates, including glycine and acetyl-CoA, serving a role as an energy metabolite [105]. Glycine is another amino acid that also feeds the one-carbon metabolism pathway, which is crucial for nucleotide synthesis and DNA and histone methylation reactions, essential processes in DNA and RNA synthesis and regulation of gene expression, all of which are essential for rapid cell division and cell growth [203]. Threonine-derived glycine is also a precursor to the synthesis of glutathione (GSH), a major intracellular antioxidant [204]. A study by Nishihara T. et al. found lower levels of total GSH in the FF of women with PCOS compared to women undergoing assisted reproductive technologies (ART) for tubal or male factors [205]. Thus, it is plausible to hypothesize that low threonine levels, together with the lower levels of glutathione peroxidase 4 in GCs of women with PCOS [206], may contribute to the heightened oxidative stress in the follicular microenvironment that characterises PCOS. Another interesting role of threonine is the synthesis of mucins, which are large glycoproteins heavily substituted with oligosaccharides in O-linkage to serine or threonine residues. Mucins are extensively expressed in the female reproductive tract, including in the ovaries and the oviducts. Zona pellucida glycoproteins are produced in the ovary. These have been hypothesised to contribute to sperm-egg recognition, whereas MUC1 and oviductin (MUC9) are expressed in the oviducts, potentially facilitating fertilisation and preventing ectopic pregnancies [207]. Therefore, low threonine levels in the follicular environment may compromise mucin synthesis, at least by the ovary. To sum up, we may hypothesize that lower threonine levels in the FF of women with PCOS may contribute to the metabolic dysfunction and oxidative environment, which could negatively impact oocyte development and fertility. However, further research on threonine metabolism in the follicular environment is necessary to understand its role in PCOS.

Glutamine [76, 95], arginine [67, 71, 95], and citrulline [90, 95] were also shown to be consistently lower in the FF of PCOS as compared to normo-ovulatory control women. Glutamine is a major nutrient for proliferating cells as it serves as a substrate for protein and nucleotide synthesis and anaplerotic fuel for the Krebs cycle. In the intrafollicular environment, glutamine plays an important role in GCs proliferation and estrogen production [208]. Additional evidence has further highlighted the relevance of glutamine metabolism within the follicular microenvironment in PCOS [209]. Wang L. et al. found a positive correlation between glutamine levels in the FF and isocitrate dehydrogenase 1 (*IDH1)* gene expression in GCs from women undergoing IVF, as well as with the ratio of higher-quality/cleaved embryos, suggesting a role for glutamine in oocyte and embryo quality. Using an *in vitro* approach, the same study demonstrated that glutamine (2.5 mM and 5 mM) improved GCs proliferation and enhanced estrogen secretion [208]. Moreover, glutamine supplies glutamate for glutathione (GSH) synthesis, contributing to antioxidant defences. Indeed, *in vitro* exposure of cumulus GCs to 5 mM of glutamine upregulated glutathione peroxidase (*GPX1*), and *IDH1* gene expression, both key antioxidant genes [208]. Arginine is the substrate for nitric oxide (NO) and citrulline synthesis [210]. At physiological levels, NO regulates the follicular development by promoting angiogenesis and controlling apoptosis. Furthermore, arginine and NO, through their action in the hypothalamus/pituitary, stimulate GnRH and LH secretion, enhancing follicular development and ovulation [211, 212]. Citrulline, produced alongside NO, can be recycled back to arginine through the urea cycle; thus, arginine and citrulline together sustain the NO cycle within the body (Fig. 5). The lower levels of arginine and citrulline in PCOS may reflect and exacerbate follicular oxidative stress and inflammatory environment. In addition, the low glutamine can directly impair the redox balance, by reducing glutamate supply, and thereby diminishing glutathione and NADPH synthesis (via IDH1). Similarly, low intrafollicular arginine may impair NO-mediated antioxidant capacity. NO can act as an antioxidant molecule, scavenging superoxide and limiting lipid peroxidation [210], so low arginine levels can potentially lead to decreased NO production and increased ROS levels. Moreover, citrulline levels were found to be lower in the FF of women with PCOS and overweight, as compared to women with PCOS and normal-weight [95]. This suggests that PCOS and body corpulence could have a synergistic effect when contributing to lower citrulline levels in the FF. Therefore, citrulline, together with the FFA C16:0, represents more than a useful biomarker for differentiating metabolic phenotypes within PCOS; it also warrants further investigation to clarify its role and contribution to the pathogenesis of the condition. Collectively, the overall lower glutamine, arginine and citrulline levels in the FF of women with PCOS suggest impaired energy and redox homeostasis, which may contribute to higher ROS levels, altered steroidogenesis and poor oocyte quality observed in this condition.

### Mitochondrial metabolism-related metabolites

High levels of succinate and malate, both Krebs cycle intermediates, also appear to characterise the follicular environment of women with PCOS [76, 87, 97], suggesting impaired cellular energy metabolism. Alongside glycolysis, the Krebs cycle regulates the cell’s energy homeostasis. Malate was also reported to be elevated in the FF of women with PCOS compared to normo-ovulatory controls by two independent studies, suggesting impaired Krebs cycle activity and possible mitochondrial dysfunction [87, 97]. Zhao H. et al. further analysed PCOS subgroups and found that high levels of malate, succinate, and oxaloacetate were linked to the hyperandrogenic phenotype but not to obesity, since no differences were found in the levels of these three metabolites between women with PCOS with or without obesity [97]. These findings are further supported by Hou E. et al. and Sun Y. et al., both of which reported higher succinate or malate levels in the FF of women with PCOS compared to normo-ovulatory controls, despite having no significant BMI differences between the groups [76, 87]. In the study by Hou E. et al., the higher succinate levels in the FF were accompanied by a decrease in succinate dehydrogenase activity – the enzyme that converts succinate into fumarate with the production of FADH2 (also known as complex II of the mitochondrial electron transport chain), implying Krebs Cycle and electron transport chain dysfunction in PCOS [76]. These findings suggesting mitochondrial dysfunction were further corroborated by Zhao H. et al. [97], who reported alterations in the distribution and morphology of mitochondria within the cumulus cells of women with PCOS, as well as lower mitochondrial membrane potential, reduced mitochondrial biogenesis, and increased mitophagy. Elevated ROS and oxidative stress levels, marked by higher ratios of NAD^+^/NADH and NADP^+^/NADH, were also observed in cumulus cells of women with PCOS. Of note, high succinate levels in the follicular microenvironment may be both a consequence of impaired mitochondrial function, as well as a contributing factor, through their regulatory role in DNA and histone methylation [213]. Higher succinate levels may increase the methylation ratio of peroxisome proliferator-activated receptor gamma coactivator 1-alpha (PGC-1α) promoter, resulting in decreased PGC-1α mRNA levels and subsequent diminished mitochondrial biogenesis. Elevated succinate levels can also be derived from glutamine through glutamine-dependent anaplerosis and the γ-aminobutyric acid (GABA) shunt pathway, as observed in inflammatory macrophages [214]. In these inflammatory conditions, succinate acts as a pro-inflammatory signal, increasing hypoxia-inducible factor-1α (HIF-1α) activity and interleukin-1β (IL-1β) expression. Thus, it is plausible that the higher succinate levels observed in the FF of women with PCOS may contribute to perpetuating the chronic inflammatory state associated with this condition and affect oocyte development due to its pro-inflammatory role. Reinforcing the narrative of dysfunctional mitochondria, Coenzyme Q10 was also found to be lower in the FF of women with PCOS compared to normo-ovulatory controls, although data are somewhat inconsistent [67, 75, 79]. Liu L. et al. and Lazzarino G. et al. observed lower Coenzyme Q10 levels in the FF of women with PCOS, whereas He Q. et al. reported the opposite. These discrepancies may be due to the heterogeneity of the condition and differences between the populations included in each study, as no information regarding androgen levels and IR is reported in the studies performed by Liu L. et al. and Lazzarino G. et al. Also, the relatively small population size in the study of He Q. et al., which included only six women with PCOS and six normo-ovulatory controls, could be underpowered and may not be fully representative of the PCOS and control populations. Coenzyme Q10, also called ubiquinone, is a fat-soluble molecule that transports the electrons from complexes I and II to complex III within the mitochondrial electron transport chain [215]. In its fully reduced form, ubiquinol is also a potent lipid-soluble antioxidant that protects the cells against oxidative stress. Coenzyme Q10 mediates inflammatory responses, as it modulates the expression of several genes from the signalling pathways of G-protein coupled receptors, JAK/STAT, and Integrin. Using *in silico* and *in vitro* studies, Schmelzer C. et al. proposed that Coenzyme Q10 exerts its anti-inflammatory properties by regulating several inflammatory mediators via the transcription factor NFκB1 [216]. To the best of our knowledge, the studies included in this review are the only reports of Coenzyme Q10 levels in PCOS, as there are no reports regarding Coenzyme Q10 levels in the blood of women with PCOS. However, we may hypothesize that together with the reduced activity of mitochondrial complex II reported by Hou E. et al., these results suggest decreased activity of the mitochondrial electron transport chain in PCOS. Interestingly, several studies have evaluated the effect of Coenzyme Q10 supplementation in women with PCOS, describing improvements in metabolic and hormonal endpoints. A meta-analysis by Zhang T. et al. demonstrated that Coenzyme Q10 supplementation significantly improved IR by lowering HOMA-IR, fasting insulin, and fasting plasma glucose levels; improved sex hormone levels by increasing FSH and decreasing testosterone levels; and improved blood lipids by lowering TGs, total cholesterol, and low-density lipoprotein cholesterol (LDL-C) in women with PCOS [217]. In a randomized placebo-controlled trial, women with PCOS and overweight or obesity treated with 200 mg of Coenzyme Q10 per day showed a significant decrease in circulating levels of tumour necrosis factor-α (TNF-α), high sensitivity C-reactive protein (hs-CRP), IL-6, vascular cell adhesion molecule-1 (VCAM-1), and E-selectin, demonstrating that Coenzyme Q10 improved inflammation and endothelial dysfunction in this population [218]. Another randomized clinical trial assessing the effects of Coenzyme Q10 and clomiphene citrate combination therapy as compared to clomiphene citrate alone in the fertility outcomes of women with PCOS, showed that the combination therapy group had higher ovulation (70% vs 19%) and conception rates per cycle (48.6% vs 6.3%) compared to those treated with clomiphene alone, with women with PCOS without obesity achieving even greater benefits [219]. In support of these results, our enrichment analysis also pointed towards the Krebs cycle as one of the most affected pathways in PCOS (Fig. 3). Overall, these findings highlight mitochondrial dysfunction and impaired energy metabolism as key features of PCOS. Further studies focusing on the mechanisms underlying altered Krebs cycle metabolites in PCOS, as well as the potential value of succinate as a biomarker of PCOS, would be of great interest. Also, identifying molecular targets for restoring mitochondrial function could be a promising therapeutic approach to improve the metabolic and reproductive function of women with PCOS.

### Nucleotide metabolism-related metabolites

Purine and pyrimidine metabolism also seems to be altered in PCOS, due to the consistent increase in the levels of hypoxanthine and uracil observed in the FF of women with PCOS compared to normo-ovulatory women [67, 76]. Hypoxanthine is a purine metabolite and has been implicated in oocyte arrest at prophase I in mice and other mammals [220–222]. It achieves this by inhibiting phosphodiesterase, thus preventing cAMP breakdown and maintaining the high intracellular cAMP levels required to keep the oocyte meiotically arrested until the pre‑ovulatory phase [223]. Uracil is a pyrimidine released during RNA turnover and DNA repair. Under normal conditions, uracil is salvaged or further catabolized to maintain nucleotide balance (Fig. 5). However, when its generation outpaces salvage‑pathway capacity, or when DNA repair processes excise damaged bases, uracil can accumulate [224, 225]. There is limited data concerning hypoxanthine and uracil in the intrafollicular microenvironment; however, some studies have also reported elevated hypoxanthine in the blood of women with PCOS [186, 226], while elevated urinary uracil levels were reported in another, suggesting this to be a systemic metabolic shift that characterises PCOS. Other nucleotide-metabolism-related metabolites have also been associated with PCOS, as Gong Z. et al. reported higher serum uric acid (the end-point of purine metabolism, downstream of hypoxanthine) in women with PCOS compared to normo-ovulatory controls [227]. Moreover, since in the studies by Hou E. et al. and Lazzarino G. et al. there were no differences in BMI between women with PCOS and normo-ovulatory controls, this suggests that elevated hypoxanthine and uracil in the FF is a PCOS feature rather than being associated with obesity [67, 76]. However, as IR and hyperandrogenism were not assessed in the study by Lazzarino G. et al., it remains unclear whether these factors could have influenced the levels of these metabolites.

Interestingly, levels of N^1^-methyl-4-pyridone-3-carboxamide (4PY), a terminal metabolite of nicotinamide adenine dinucleotide (NAD⁺) metabolism, were found to be lower in the FF of women with PCOS compared with normo-ovulatory controls [93, 97]. As 4PY reflects downstream nicotinamide and NAD⁺ metabolic flux, its reduction may indicate altered intrafollicular nicotinamide handling and impaired NAD⁺ turnover rather than enhanced NAD⁺ catabolism (Fig. 5). Given that the ovarian follicle represents a locally regulated metabolic compartment, decreased 4PY may further suggest compromised oxidative and/or mitochondrial capacity within GCs, a feature consistently reported in PCOS. In line with this interpretation, NAD⁺ deficiency has been implicated in impaired GCs function and associated with PCOS, supporting the presence of a localized disruption of NAD⁺ homeostasis within the ovarian microenvironment [228–230].

Overall, the data suggest systemic elevation of purine and pyrimidine metabolites in PCOS, and further studies on the potential role of these metabolites and metabolic pathways in PCOS are warranted.

### Pathway enrichment analysis

The alterations observed at the individual metabolite level in the FF of women with PCOS align closely with the pathway enrichment results, providing an additional layer of validation and reinforcing the biological coherence of these findings. The accumulation of specific glycerophospholipids, glycerolipids, and fatty acids, including both saturated and unsaturated lipid species, mirrors the enrichment of lipid metabolism pathways, supporting the hypothesis of altered membrane composition, lipid signalling, and oxidative stress in PCOS that are translated into the follicular environment. Similarly, differences in branched-chain and other amino acids correspond to the dysregulated biosynthesis and degradation pathways, highlighting compromised amino acid availability and altered cellular signalling in PCOS. Changes in Krebs cycle intermediates further validate the enrichment of energy-related pathways, suggesting altered mitochondrial metabolism, impaired mitochondrial function, and reduced bioenergetic support for oocyte maturation in PCOS. Enrichment of steroid hormone biosynthesis pathways further integrates these metabolic disturbances, consistent with altered steroidogenic balance in PCOS and its close dependence on lipid substrates and mitochondrial function. Collectively, these findings support the hypothesis that convergent disruptions in lipid, amino acid, and energy metabolism underlie the distinctive metabolic signature of the FF of women with PCOS and may contribute to impaired oocyte competence.

### Translational potential and future directions

The FF metabolomics holds considerable potential for advancing reproductive medicine, particularly in the context of PCOS. By characterizing the metabolic composition of the follicular microenvironment, metabolomic profiling offers critical mechanistic insights into ovarian-specific metabolic processes while simultaneously capturing the imprint of systemic metabolic disturbances that characterize PCOS at the follicular level. This dual perspective is especially relevant given the marked phenotypic heterogeneity of PCOS and the current limitations in understanding how systemic metabolic dysfunction translates into ovarian-specific alterations underlying follicular dysfunction and arrest, and compromised oocyte competence.

From a translational standpoint, the identification of pathway-specific metabolic alterations within the FF may contribute to a more refined understanding of how distinct PCOS phenotypes present at the ovarian level. In particular, pathways related to lipid metabolism, amino acid metabolism, redox balance, and mitochondrial function emerge as recurrently disrupted and may represent biologically relevant targets for tailored interventions aimed at restoring follicular homeostasis.

In the longer term, such metabolic signatures could inform the development of more personalized strategies to improve the clinical manifestations and reproductive outcomes of women with PCOS. Nevertheless, the clinical translation of FF metabolomics requires further validation in cohorts with well-characterized phenotypes and longitudinal studies linking metabolic profiles with reproductive function outcomes. Collectively, these findings highlight the translational potential of FF metabolomics while defining clear priorities for future research aimed at integrating ovarian-level metabolic information into phenotypic stratifications and therapeutic frameworks for PCOS management.

### Limitations

This systematic review identified the main differences in the FF metabolic profile of women with PCOS as compared to normo-ovulatory women. Despite the value of systematically synthesizing metabolomic data from the FF of women with PCOS, several limitations must be acknowledged. A major challenge lies in the inherent phenotypic heterogeneity of PCOS, which contributes to substantial variability across study populations. Notably, the majority of the studies included did not stratify women by BMI, insulin resistance status, androgen levels, or phenotype, thereby limiting the ability to associate specific metabolic alterations with distinct PCOS subtypes. This fact also emphasises the need for future metabolomic studies to systematically incorporate detailed phenotype stratification of women with PCOS to enable more reliable identification of phenotype-specific metabolic signatures. Furthermore, many studies have a small sample size, which reduces statistical power and may lead to overfitting or false associations. Additionally, inconsistencies in PCOS diagnostic criteria across studies introduce an additional source of variability. A notable limitation is that, in all of the studies included in this review, the FF was obtained after ovarian stimulation for IVF. Since exogenous gonadotropins can alter the FF metabolic profile, these findings may not entirely reflect the events observed during a natural cycle. Moreover, it remains unclear to what extent the effects of controlled ovarian stimulation per se or different pharmacological protocols used could differ between women with or without PCOS. Furthermore, since FF composition varies with the developmental stage of the follicle, the findings primarily reflect pre-ovulatory conditions, limiting insight into earlier stages of folliculogenesis. For ethical reasons, some of these limitations are difficult to avoid, namely to collect FF in conditions other than during IVF procedures. Additionally, the technical variability among the metabolomic studies must also be considered. Differences in sample preparation protocols, extraction methods, metabolites separation techniques (including the use of different LC columns), and the choice of the metabolomics platform used (LC–MS, GC–MS, or NMR) introduce biases that inevitably influence the metabolites analysed in each study. These methodological differences contribute to the heterogeneity of the results across studies. Furthermore, metabolomic analyses are inherently hypothesis-generating and exploratory by nature, rather than conclusive, and should therefore be interpreted with appropriate caution. Correspondingly, the findings from metabolomic analysis require follow-up validation studies of specific metabolites identified to elucidate the underlying molecular mechanisms, as well as their biological implications or their utility as diagnostic biomarkers. Regarding the systematic review method itself, it is also important to note that some relevant studies may have been missed due to limitations in search strategy, keyword choice, or full-text availability.

## Conclusion

In this systematic review, we have summarized the current evidence on the FF metabolomic profile that distinguishes women with PCOS from normo‑ovulatory controls. Across studies, steroid hormone precursors and intermediates were consistently different, reflecting significant shifts in steroidogenesis. FF from women with PCOS also exhibited higher levels of saturated, monounsaturated, and polyunsaturated fatty acids, in support of lipotoxic stress as a contributor to impaired follicular development. Phospholipid profiling also revealed significant shifts in PC and PE species, suggesting altered membrane dynamics and signalling. Furthermore, branched‑chain amino acids, energy‑metabolism intermediates, and nucleotide‑related metabolites were consistently found to be higher in the FF of women with PCOS, highlighting the putative dysfunction in bioenergetics and nucleotide turnover. All together, these metabolic fingerprints reveal fundamental derangements in lipid handling, steroidogenesis, and bioenergetics within the follicular niche, which underlie the reproductive dysfunction of women with PCOS. By pinpointing specific metabolites and pathways that consistently differentiate the FF of women with PCOS from normo-ovulatory controls, our review provides insights for future multi‑omics and mechanistic studies, as well as opening new avenues of research to validate the use of these metabolites as diagnostic biomarkers or treatment targets. Taken together, these findings support FF metabolomics as a valuable framework for elucidating ovarian-level metabolic heterogeneity in PCOS and for guiding future mechanistic and translational studies aimed at developing targeted strategies for delivering personalized care and improving reproductive outcomes of women with PCOS.

## Supplementary Information

Below is the link to the electronic supplementary material.

Supplementary file1 (XLSX 81 KB)

Supplementary file2 (PDF 366 KB)

## Data Availability

No datasets were generated or analysed during the current study.

## Abbreviations

4PY: N1-Methyl-4-pyridone-3-carboxamide
17-OHP4: 17-Hydroxyprogesterone
ACTH: Adrenocorticotropic Hormone
AMH: Anti-Müllerian Hormone
ART: Assisted Reproductive Technologies
AUC: Area Under the Curve
BCAAs: Branched-Chain Amino Acid
BMI: Body Mass Index
β-oxidation: Beta-oxidation
FAI: Free Androgen Index
FF: Follicular Fluid
FFAs: Free Fatty Acids
FDR: False Discovery Rate
FSH: Follicle-Stimulating Hormone
GABA: Gamma-Aminobutyric Acid
GCs: Granulosa Cells
GnRH: Gonadotropin-Releasing Hormone
GPCRs: G Protein-Coupled Receptors
GPX1: Glutathione Peroxidase 1
GSH: Glutathione
GWAS: Genome-Wide Association Studies
HA: Hyperandrogenism
HOMA-IR: Homeostasis Model Assessment of Insulin Resistance
HPO: Hypothalamic-Pituitary-Ovarian (axis)
IR: Insulin Resistance
IVF: *In Vitro* Fertilization
KEGG: Kyoto Encyclopedia of Genes and Genomes
LH: Luteinizing Hormone
LPA: Lysophosphatidic Acid
LPC: Lysophosphatidylcholine
LPE: Lysophosphatidylethanolamine
MUFAs: Monounsaturated Fatty Acids
NAD^+^: Nicotinamide Adenine Dinucleotide
OA: Oligo- or Anovulation
OHSS: Ovarian Hyperstimulation Syndrome
PC: Phosphatidylcholine
PCOM: Polycystic Ovarian Morphology
PCOS: Polycystic Ovary Syndrome
PE: Phosphatidylethanolamine
PGE2: Prostaglandin E2
PUFAs: Polyunsaturated Fatty Acids
ROC: Receiver Operating Characteristic
ROS: Reactive Oxygen Species
SCD: Stearoyl-CoA Desaturase
SFAs: Saturated Fatty Acids
SHBG: Sex Hormone-Binding Globulin
StAR: Steroidogenic Acute Regulatory Protein
TG: Triglyceride
TNF-α: Tumor Necrosis Factor Alpha
